# Mosaic dental morphology in a terminal Pleistocene hominin from Dushan Cave in southern China

**DOI:** 10.1038/s41598-019-38818-x

**Published:** 2019-02-20

**Authors:** Wei Liao, Song Xing, Dawei Li, María Martinón-Torres, Xiujie Wu, Christophe Soligo, José María Bermúdez de Castro, Wei Wang, Wu Liu

**Affiliations:** 1 State Key Laboratory of Geological Processes and Mineral Resources, School of Earth Sciences, China University of Geosciences, Wuhan, 430074 China; 2 Anthropology Museum of Guangxi, Nanning, 530028 Guangxi China; 3 Key Laboratory of Vertebrate Evolution and Human Origins of Chinese Academy of Sciences, Institute of Vertebrate Paleontology and Paleoanthropology, Chinese Academy of Sciences, Beijing, 100044 China; 4 CAS Center for Excellence in Life and Paleoenvironment, Beijing, 100044 China; 5 Department of Anthropology, University College London (UCL), 14 Taviton Street, London, WC1H 0BW UK; 6 National Research Center on Human Evolution (CENIEH), Paseo Sierra de Atapuerca s/n, Burgos, 09002 Spain; 7 Institute of Cultural Heritage, Shandong University, 72 Jimo-Binhai Road, Qingdao, 266237 China

## Abstract

Recent studies reveal high degrees of morphological diversity in Late Pleistocene humans from East Asia. This variability was interpreted as complex demographic patterns with several migrations and possible survival of archaic groups. However, lack of well-described, reliably classified and accurately dated sites has seriously limited understanding of human evolution in terminal Pleistocene. Here we report a 15,000 years-old *H. sapiens* (Dushan 1) in South China with unusual mosaic features, such as large dental dimensions, cingulum-like structures at the dentine level in the posterior dentition and expression of a “crown buccal vertical groove complex”, all of which are uncommon in modern humans and more typically found in Middle Pleistocene archaic humans. They could represent the late survival of one of the earliest modern humans to settle in an isolated region of southern China and, hence, the retention of primitive-like traits. They could also represent a particularity of this group and, hence, reflect a high degree of regional variation. Alternatively, these features may be the result of introgression from some late-surviving archaic population in the region. Our study demonstrates the extreme variability of terminal Pleistocene populations in China and the possibility of a complex demographic story in the region.

## Introduction

As a key region of Late Pleistocene human evolution, East Asia has for the last ten years been the source of remarkable new insights into the origin, evolution and interaction of modern humans. A series of recent studies revealed that the morphology and evolutionary patterns of Late Pleistocene humans in East Asia are more complicated than previously documented^1–3^. The discovery and study of the Late Pleistocene hominin fossils of the Huanglong Cave, Zhirendong, Daoxian and other sites in Southern China indicate that modern humans appeared in the region as early as 120–80 ka^4–7^ increasing the time of possible overlap with other archaic hominins. The study of the hominin fossils recovered at Xujiayao and the new findings from the Xuchang site reveal that in the transition from the late Middle to the early Late Pleistocene North China was inhabited by some primitive populations with unclear taxonomic assignment^8–10^. The fossil discoveries and related studies of *H. floresiensis*^11,12^ at Liang Bua Cave on Flores, Indonesia, and the fossil and genetic data recovered at Denisova Cave in southern Siberia^13,14^ further support the late survival of some archaic populations that likely overlapped in time and geography with modern humans in East Asia. In addition, the genetic analysis of the Denisovans and the individual from the Tianyuan Cave revealed possible interbreeding of these hominins with Neanderthals, early modern humans or even archaic populations in or around Oceania^14–16^.

In the general context of a taxonomically more diverse scenario, the morphology and evolution of hominins in the late part of the Late Pleistocene, in particular, have been the subject of increasing attention. Both morphological and genetic analyses of the populations that inhabited East Asia during this period reveal a high diversity that has been interpreted as evidence of a coexistence of more than one hominin lineage and/or the interbreeding between hominin groups^15–18^. Recent analyses of both mitochondrial and Y chromosome DNA revealed high levels of genetic diversity in the populations of southern China, especially in Southwest China, with persistence of some novel basal haplogroups that directly emanate from the Eurasian founder nodes. Subsequent analyses suggested that these newly identified basal lineages likely represent the genetic relics of modern humans initially peopling East Asia instead of being the results of gene flow from the neighboring regions. This diversity was caused by either novel indigenous haplogroups or “foreign” lineages introduced by the ancient and/or recent migration event(s). Southwest China was likely the genetic reservoir of modern humans after they entered East Asia^19,20^. According to some researchers, the morphology of the terminal Pleistocene human fossils from Maludong and Longlin, both in Southwest China could be indicative of complex demographic patterns, with several human migrations and possible survival of archaic populations^21–23^ although their relationship with other Late Pleistocene-Holocene populations from mainland Southeast Asia remains unclear^24,25^. In addition, the identification of ancient mt-DNA and Y-DNA lineages in Southwest China has led to the definition of this region as a “hotspot of human diversity”^19,20,23^. Despite these advances, the detailed morphological pattern, diversity and evolutionary implications of the terminal Pleistocene hominins in Southern China need further investigation. The lack of a substantial number of well-described, reliably classified and accurately dated sites has seriously limited our understanding of human evolution in the Pleistocene-Holocene transition in southwest China. In the present study, we aim to contribute to this topic by reporting a recently discovered human dentition from Dushan Cave, southern China.

**Figure 1:**
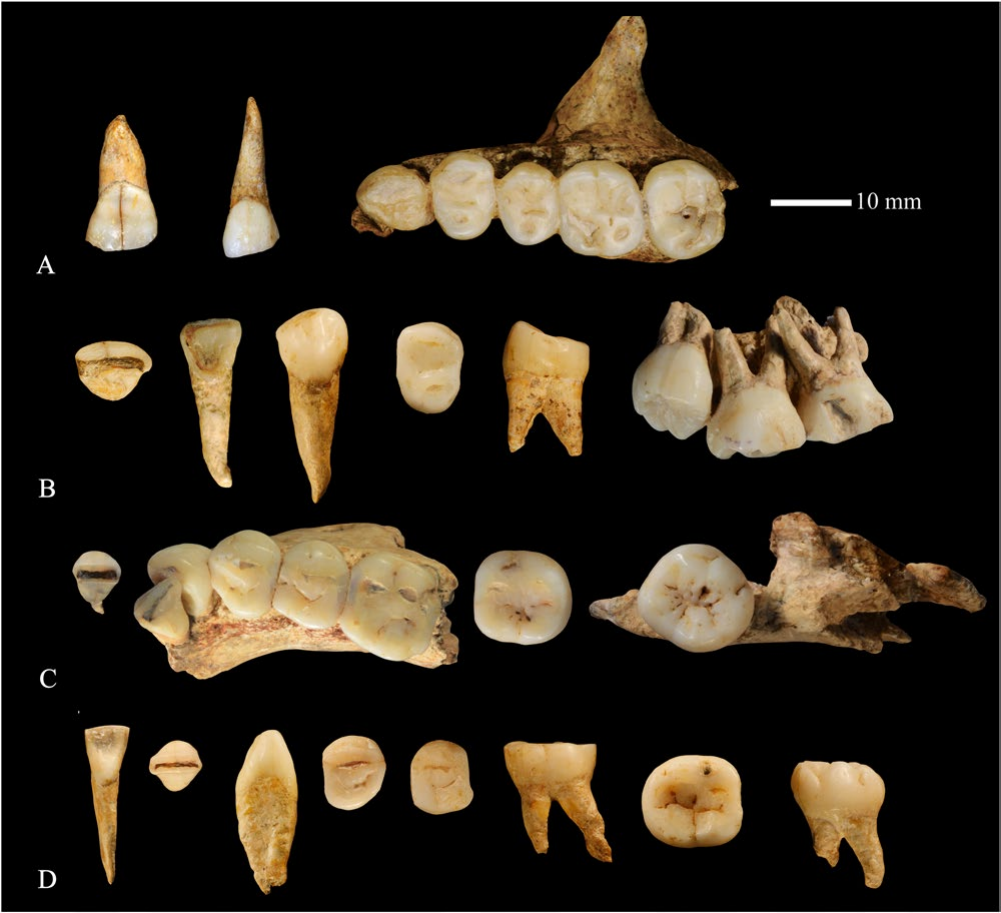
The maxillary and mandibular dentitions of Dushan 1. (**A**) left maxillary dentition; (**B**) right maxillary dentition; (**C**) right mandibular dentition; (**D**) left mandibular dentition. From left to right: I1 to M3. Left M^3^ was missing).

Dushan Cave is located in Linfeng Town, Tiandong County, Guangxi Zhuang Autonomous Region in South China. Excavation at Dushan Cave in 2011 unearthed some human remains, a rich assemblage of stone tools and some faunal remains (see SI-1 for details). The human fossils found at Dushan Cave include a partial cranium, mandibular fragments, and complete upper and lower dentitions except for the left M^3^ (Fig. 1, SI-Fig. 3 and SI-Table 2). All the fossils belong to the same individual (Dushan 1). AMS radio carbon dating plus geology and faunal analysis indicate that Dushan 1 lived in this area during the period from ~15,850 to ~12,765 years BP (see SI-1 for details). In the present study, the metrics and both the external (outer enamel surface, OES) and internal (enamel dentine junction, EDJ) morphology of Dushan 1 are collected and compared to a large sample of fossil and contemporary *Homo sapiens* as well as some archaic *Homo* populations from East Asia.

## Results

The analysis and comparisons of the metric and non-metric traits of Dushan 1 teeth with those of various fossil hominins and recent modern humans in the present study indicate that both the maxillary and mandibular dentitions of Dushan 1 exhibit a mosaic morphological pattern combining primitive and derived traits. Nearly all the primitive dental traits identified in Dushan 1 resemble those of Middle Pleistocene *Homo erectus* and are unusual and/or absent in *H. sapiens*. Apart from the preliminary evaluation of the skull and the mandible, Dushan 1 teeth also present some derived features that are typically found in *H. sapiens*^26–28^. Our study of the dental morphology of Dushan 1 suggests that it represents a *Homo sapiens* individual with an unusual retention of ancestral traits.

### Dental traits typical of *H. sapiens* in Dushan 1

In the present study, each individual tooth of Dushan 1 was described and compared (SI-4, SI-5, SI-Table 9) with particular attention to a selection of 69 metric and non-metric traits listed in SI-Table 10. Below we further summarize the expression of the most relevant ones (see also Table 1) pointing to affinities with modern humans.

**Table 1:** The frequency and expressions of key dental traits in Dushan 1 and comparative samples.

		Dushan 1	East Asia archaic *Homo*	East Asia EMH	East Asia RMH	Java H. erectus	Neanderthal	Global H. sapiens
**Trait comparisons (Frequency and specimen number)**								
3	I^1^ Labial convexity	1/2 (EP-2)	92.8% (13/14)	42.9% (3/7)	9.6% (8/83)		100% N = 21	30.8% N = 91
6	I^2^ Interruption groove present	1/2 (Hs)	0% (0/7)	0% (0/6)	43.6% (92/211)			10.4–65.0% N = 5693
9	P^3^ Transverse crest present	0/2 (Hs)	17.7% (3/17)	20% (1/5)	4.2% (3/71)	33.3% (3/9)	6.7% N = 15	2% (6/283)
10	P^4^ Transverse crest present	0/2 (Hs)	21.4% (3/14)	25% (1/4)	2.9% (2/70)	50% (4/8)	12.4% N = 16	2% (5/279)
13	P^3^ Bifurcated essential crest	2/2 (EP-2)	100% (15/15)	0% (0/4)	44.2% (23/52)		53.3% N = 15	11.1% N = 126
14	P^4^ Bifurcated essential crest	2/2 (EP-2)	100% (16/16)		55.3% (26/47)		78.6% N = 14	9.4% N = 106
17	P^3^ Mesial or distal accessory ridge	2/2 (Hs)	93.3% (14/15)	50% (2/4)	43.3% (26/60)		36.4% N = 11	23.4–57.0% N = 749
18	P^4^ Mesial or distal accessory ridge	2/2 (Hs)	93.8% (15/16)		67.6% (46/68)		41.7% N = 12	33.0–80.2% N = 749
19	P_3_ Mesial or distal accessory ridge	2/2 (Hs)	100% (10/10)	25% (1/4)	52.0% (40/77)		90% N = 20	11.9% N = 13521
20	P_4_ Mesial or distal accessory ridge	2/2 (Hs)	100% (8/8)	33.3% (1/3)	46.6% (34/73)		70% N = 20	23% N = 115
21	P^3^ Accessory marginal tubercle	2/2 (EP-2)	93.3% (14/15)	25% (1/4)	19.2% (10/52)			
22	P^4^ Accessory marginal tubercle	2/2 (EP-2)	93.8% (15/16)		14.9% (7/47)			
24	P^4^ Two-rooted premolars	2/2 (EP-2)	40% (2/5)	0% (0/6)	4.3% (2/47)			
27	Three-rooted P_3_	2/2 (EP-1)	0% (0/9)	0% (0/5)	0% (0/46)			0% (0/599)
28	P_3_ Tomes’s Root	2/2 (EP-2)			64.8% (142/219)			19.9% N = 1371
39	M^1^ Carabelli’s trait (Dushan 1 has special expression)	2/2 (EP-1+ EP-2)	45% (9/20)	21.4% (3/14)	9.7% (27/279)	100% (2/2)	50% N = 20	1.9–36.0% N = 2426
49	M_1_ protostylid (Dushan 1 has special expression)	2/2 (EP-1+ EP-2)	53.9% (7/13)	18.2% (2/11)	18.3% (63/345)		3.2% N = 31	18.7% N = 2362
60	Three-rooted M_1_	2/2 (Hs)	7.7% (1/13)	0% (0/15)	24.2% (15/62)			0–31.1%
62	Complex occlusal morphology at EDJ of molars	5/5 (EP-2)	100% (16/16)	15.2% (5/33)	5.2% (8/153)			
63	Buccal basal swelling C^1^- M^3^ C_1_- M_3_	2/2 2/2 (EP-2)	54.6–63.6% N = 19 55.6–88.9% N = 22	14.6% N = 82 12.9% N = 70	0–25.7% N = 101 0–12.9% N = 126			
65	Crown buccal vertical groove complex	Present (Unique + EP-1)	Absent	Absent	Absent N = 5		Weakly expressed (La Quina H18)	
66	Tooth size proportions I^1^-C^1^/P^3^-M^3^	0.32 (Hs)	0.40	0.37	0.37		0.42	0.36
67	Tooth size proportions I_1_-C_1_/P_3_-M_3_	0.26 (Hs)	0.27	0.25	0.26		0.31	0.26
68	Molar size % increase M_1_– M_2_	−0.5/+2.6 N = 2 (EP-2)	Mean = −1.4 − 11.5/+6.3 N = 6	Mean = 1.2 −3.1/+9.9 N = 8	Mean = −5.0 −14.2/+4.8 N = 174	+4/+9 N = 5		−15/+5 N = 250
69	Molar size % increase M_2_– M_3_	−1.3/−0.9 N = 2 (EP-2)	Mean = −2.8 −13.1/+6.3 N = 5	Mean = −3.7 −6.4/+4.7 N = 5	Mean = −2.6 −18.9/+18.0 N = 124	−10/0 N = 5		−15/+14 N = 211

Morphological status of Dushan Cave in the parentheses.

‘EP-1’: a primitive condition shared with the Africa *Austrapithecus* and early *Homo*.

‘EP-2’: a primitive condition shared with the Eurasia archaic *Homo*.

‘Hs’: a derived condition shared with H. sapiens.

‘Hs-Eu’: a derived condition shared with European H. sapiens or Neanderthal.

‘unique’: a unique condition restricted to Dushan Cave.

In general, both upper and lower anterior teeth of Dushan 1 are gracile and simple in their morphology (Fig. 1, SI-Figs 3, 7, 8, 10 and 11) resembling those of *H. sapiens*. Except the upper central incisors, all other incisors of Dushan 1 (I^2^s, I_1_s and I_2_s) exhibit weakly developed double shoveling which is usually regarded as autapomorphic of *H. sapiens*^26–28^. The interruption groove found in the I^2^ of Dushan 1 is also a typical modern human feature^27^ and to our knowledge, it has not been found in any Middle Pleistocene or Late Pleistocene *Homo* from East Asia but is particularly frequent in modern humans in this region (Table 1).

All the upper and lower premolars of Dushan 1 exhibit well-developed mesial or distal accessory ridges (Fig. 3, Table 1, SI-Table 10). This feature is found in nearly all the Middle Pleistocene *Homo* specimens from East Asia and Neanderthals. In Late Pleistocene early modern humans, the frequencies of mesial or distal accessory ridges in both upper and lower premolars decrease to about 50%. Our observations indicate that 43–67% of upper and lower premolars of East Asian recent modern humans (RMH) have this feature. The frequencies of this trait in global RMH populations reported by Burnett and colleagues^29^ are 23.4–57.0% and 33.0–80.2% for P^3^ and P^4^, respectively. The frequencies they report for East Asian populations are even higher (50.7–80.2%). Considering both fossil hominins and recent modern humans have higher frequencies of the accessory ridge in premolars, we tentatively consider the occurrence of this trait in the premolars of Dushan 1 as resemblance to *H. sapiens*.

In addition, there are other traits in the postcanine dentition of Dushan 1 that are more frequently observed in *H. sapiens* such as the lack of transverse crests in both upper and lower premolars^30,31^ or the three-rooted M_1_, a typical *H. sapiens* feature with higher frequencies in East Asian populations^27,32^.

### Archaic dental traits in Dushan 1

In addition to the modern dental traits mentioned above, Dushan 1 upper and lower dentitions display more archaic traits that are not usually found in modern humans (see Table 1 for details).

#### Crown size

**Figure 2:**
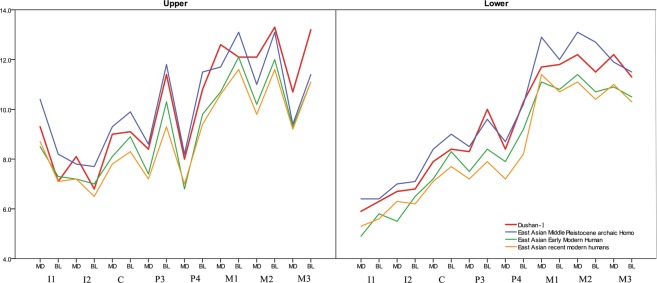
Mesiodistal (MD) and buccolingual (BL) dimensions of Dushan 1 teeth and comparative samples from East Asian Pleistocene *Homo* and recent human populations.

The crown sizes of Dushan 1 upper and lower teeth are very large compared to those of Middle Pleistocene archaic *Homo*, early modern humans and recent modern humans from East Asia. All the mesiodistal (MD) and buccolingual (BL) crown dimensions of Dushan 1 (except for the BL dimensions of I^1^ and I^2^) exceed the diameters of Late Pleistocene early modern humans and Holocene modern humans and, in some dimensions, they are close to or even exceed the values of Asian Middle Pleistocene archaic *Homo* (Fig. 2 and SI-Table 10).

Relative tooth size differences between Dushan 1 individual and comparative samples including Neanderthals, early and recent modern humans from China, the Levant, North Africa and the Canary Islands, are further confirmed by Principal Component Analysis (PCA) of crown dimensions across dental classes in both upper and lower dentition (see SI-4 for details). Indices of tooth size proportions between anterior and posterior teeth also exhibit a similar trend (Table 1 and SI-Table 10), and reveal the resemblance of Dushan 1 to modern humans in its anterior teeth^18^. Additional analysis of both bivariate plots and PCA of the bucco-lingual widths of upper and lower I2, P3, and M1 of Dushan 1 and some specimens that preserve the three dental classes also reveal the same trend (see SI-4 for details).

#### Molar size proportions

Previous studies indicate a marked size reduction of the molar series during the course of evolution of the genus *Homo*, which caused the molar size sequence to change from a primitive pattern of ‘M1 < M2 < M3’ to a derived pattern of ‘M1 > M2 > M3’^33,34^. In the present study, we compare the lower molar crown size proportions (square root of the calculated crown area [MD × BL]) from M_1_ to M_2_ ([M_2_ − M_1_]/M_1_), and from M_2_ to M_3_ ([M_3_ − M_2_]/M_2_) in Dushan 1 and comparative samples following the approach of Kaifu^31^.

As shown in Table 1, there is only a slight size increase or decrease from M_1_ to M_2_ (−0.5% to +2.6%) and from M_2_ to M_3_ (−1.3% to −0.9%) in Dushan 1. Compared to those of the fossil hominins and recent modern humans (Table 1, SI-Table 10), the molar size portions of Dushan 1 exhibit the primitive status relative to the Late Pleistocene early modern humans and resemble Middle Pleistocene archaic *Homo*.

#### I^1^ Labial convexity

**Figure 3:**
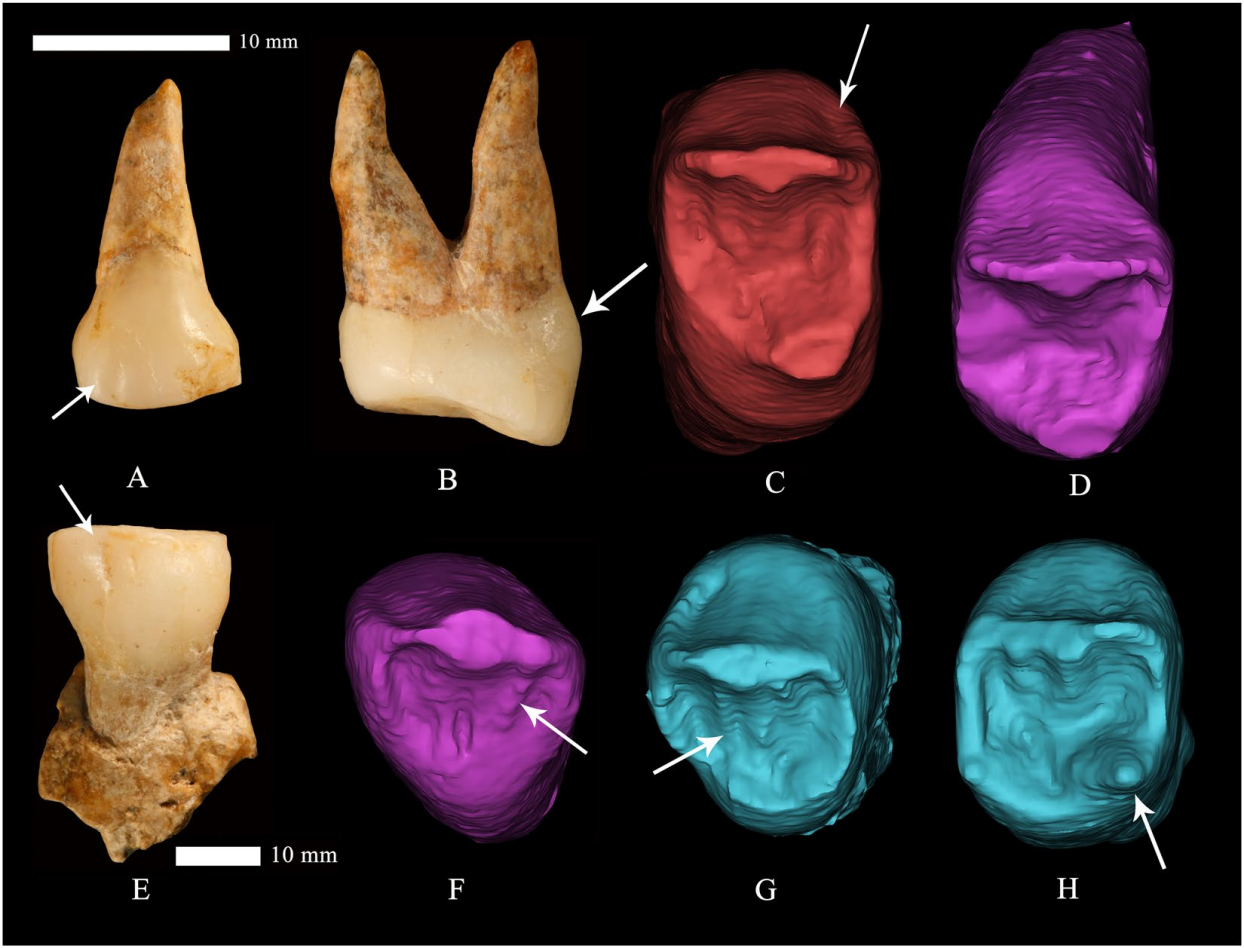
Some morphological traits identified in Dushan 1 teeth. (**A**) buccal view of right P^4^. Arrow indicates the buccal vertical groove. (**B**) Distal view of right P^3^ showing the divergent roots and basal bulging. (**C**) Occlusal view of right P^3^. (**D**) Occlusal view of left P^4^. (**E**) Buccal view of left P_3_. Arrow indicates the buccal groove. (**F**) Occlusal view of left C^1^. (**G**) Occlusal view of right P_3_. (**H**) Occlusal view of left P_4_. Note the pronounced crown buccal vertical grooves, basal bulging and complicated occlusal morphologies in all teeth. (All the EDJ images are not scaled).

Moderate to pronounced labial convexity occurs more often in earlier hominins, including Middle Pleistocene hominins and Neanderthals (Table 1, SI-Table 10)^27,30,33^. *H. sapiens* is the only group that displays completely flat labial surfaces (ASUDAS grade 0), although early *H. sapiens* such as Qafzeh or Dolni Vestonice may show labial surfaces with more pronounced grades than contemporary humans. For East Asian Pleistocene hominins, the frequencies of the I^1^s labial convexity decreased from 92.8% (Middle Pleistocene archaic *Homo*) to 42.9% (early modern humans) (≥grade 2). Our observation shows the left and right I^1^s of Dushan 1 exhibit weak and trace labial convexity respectively (ASUDAS grades 2 and 1). But more pronounced labial convexities are also identified in the C^1^s of Dushan 1 (ASUDAS grade 4) (Fig. 1, SI-Figs 3, 7 and 8).

#### Bifurcated essential crest (P^3^and P^4^)

The bifurcated essential crests for P^3^ and P^4^ are more frequently observed in earlier hominins^30,35,36^. Our data for East Asian Pleistocene hominins show that bifurcated essential crests of P^3^ and P^4^ only occurred in Middle Pleistocene archaic *Homo*. Bifurcated essential crests of upper premolars are also found in some Middle Pleistocene populations from Europe (like Atapuerca SH) and Neanderthals^30^. In our small sample of recent modern Chinese humans (52 and 47 for P^3^ and P^4^, respectively), bifurcated essential crest can be observed in 44.2% and 55.3% of the specimens (Table 1, SI-Table 10), while the frequencies of this feature in the P^3^ and P^4^ of global recent modern humans are only 11.1% and 9.4% respectively^30^. Therefore, the presence of bifurcated essential crests in both P^3^ and P^4^ of Dushan 1 (Fig. 3) may represent a primitive condition or a feature particularly more frequent in China since early times.

#### P^3^ and P^4^ accessory marginal tubercle

Over 90% of East Asian Middle Pleistocene *Homo* specimens exhibit accessory marginal tubercles in their upper premolars. The occurrence of this feature in P^3^s of early modern humans is only 25%. The frequencies of accessory marginal tubercles in upper premolars of recent modern humans are even lower (19.2% and 14.9% respectively). This feature can also be observed in Dmanisi and Middle Pleistocene European hominins. All the Dmanisi upper premolar (P^3^ of D3672 and two P^4^s of D2700 and D2282) have their central grooves bifurcating before reaching the marginal ridges resulting in mesial and distal accessory tubercles^37^. The accessory marginal tubercles are observed in 94.3% of the Atapuerca SH upper premolars^30^. Thus, the presence of accessory marginal tubercles in Dushan 1 P^3^ and P^4^ can be regarded as a primitive feature (Fig. 3, Table 1, SI-Table 10).

#### Upper molars Carabelli’s trait

**Figure 4:**
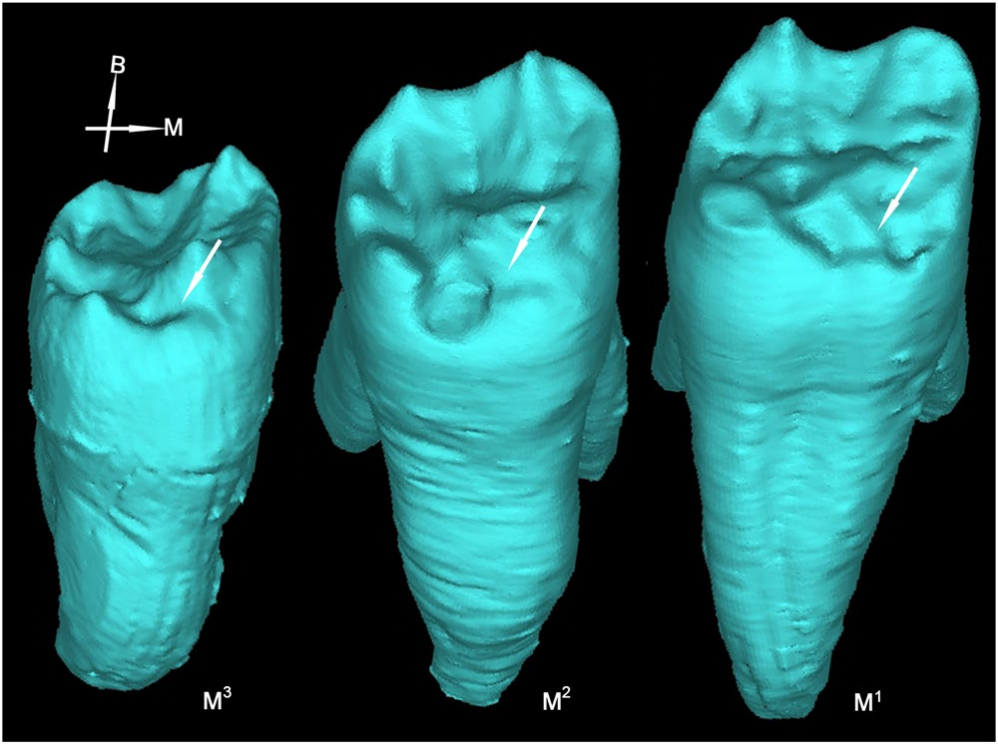
Lingual view of Dushan 1 right upper molars showing cingulum-like Carabelli’s cusps.

All the five upper molars of Dushan 1 exhibit Carabelli’s trait with more pronounced expressions at EDJ level (Fig. 4). Carabelli’s trait frequencies have high intra- and inter-population variability so the taxonomic utility of its prevalence is limited^33^. However, there are some important differences in the pattern of expression of Carabelli’s trait^38,39^. In general, even in the most pronounced cases, Carabelli’s trait in modern humans still express as the shape of a cusp that can sometimes express a free apex. However, in earlier fossil hominins and in non-human primates, Carabelli’s trait tends to adopt the shape of a cleft or cingulum-like feature that commonly exceeds the protocone surface^30,36–40^. It has been suggested that Carabelli’s trait is a cingulum remnant on hominin upper molars^38,39^. The frequencies of occurrence of Carabelli’s trait in modern human populations are low, ranging between 1.9–36.0% with 9.7%^27,33^ in East Asian RMH (Table 1, SI-Table 10).

The Carabelli’s trait expression is one of the most remarkable features of Dushan 1 teeth. All the upper molars of Dushan 1 have a deep and pronounced groove running along the upper part of the lingual side, near the occlusal edge of the protocone and the hypocone. Carabelli’s trait is also found in Chinese Middle Pleistocene *Homo erectus* of Hexian, Zhoukoudian and Yiyuan. The Carabelli’s trait all express as pits, Y-shaped depressions or small cusps in these specimens (SI-Fig. 13). They are limited to the lingual surface of the protocone, and are not as pronounced or uniquely expressed as in Dushan 1^41,42^. In Javanese *Homo erectus*, Carabelli’s trait usually weakly express as narrow pits or Y-shaped depressions (grade 4 in Ortiz’s standard)^43,44^. It is noteworthy that the expression of Carabelli’s trait in Dushan 1 strongly resembles that of the late-Middle or early-Late Pleistocene archaic human from Xujiayao. The Xujiayao M^1^ and M^2^ (PA1480-5 and PA1480-6) also exhibit the horizontal groove-like or shelf-like Carabelli’s cusp complex involving both protocone and hypocone (SI-Fig. 13)^8^. A recent study of the Denisovan M^2^ (Denisova 4) shows that this tooth exhibits a similar Carabelli’s trait expression with a massive horizontal groove with multiple apexes occupying the whole base of the protocone and crossing the mesio-lingual groove. This expression pattern was regarded as a manifestation of rudimentary derivatives of the cingulum^45^. Carabelli’s trait in *Australopithecus* is more often expressed as a composite feature formed by the lingual cingulum and wrinkled enamel associated with one or more cuspules, which usually involves the lingual surface of both protocone and hypocone. The lingual surface of *A. africanus* upper molars generally exhibits a partial or complete shelf-like cingulum, whereas that of P. *robustus* more frequently possesses a deep but small pit and/or one or more shallow grooves^38–40^.

The shelf-like Carabelli’s cusp extending across the lingual surface of both the protocone and the hypocone in Dushan 1 differs from that of most Asian Middle Pleistocene hominins but resembles that of the Chinese archaic hominin from Xujiayo and possibly Denisovans. We have not found a shelf-like Carabelli’s trait in any *H. sapiens* upper molars. However, the scarcity of EDJ data for a wide and geographically comprehensive *H. sapiens* sample obliges us to be cautious about its interpretation. Overall, we consider that the expression of a “shelf-like Carabelli’s trait” in Dushan 1 suggests retention of a primitive feature.

#### Protostylid

**Figure 5:**
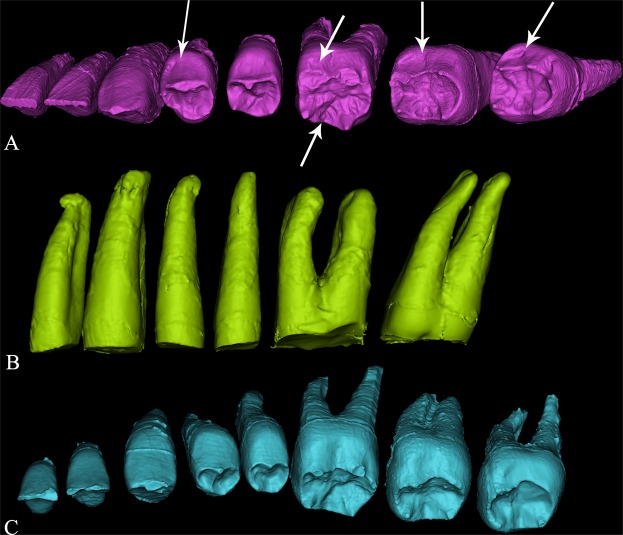
Buccal EDJ views of right lower dentitions for Dushan 1 (**A**), Tianyuan Cave (only I_2_-M_2_ available) (**B**) and modern human. (**C**) The arrows indicate the pronounced buccal vertical groove, cingulum-like protostylid and complicated occlusal morphologies. (All the EDJ images are not scaled).

Just as Carabelli’s trait in upper molars, the protostylid is also regarded as a cingular derivative^33,46–48^. All lower molars of Dushan 1 exhibit a pronounced protostylid, which is more pronounced still at the EDJ surface (Fig. 5, SI-Fig. 14). Early hominins usually have higher frequencies and more pronounced expressions of this trait. About 50% of the *Australopithecus* and early *Homo* members of Africa show this feature^46–48^. East Asian Middle-Late Pleistocene archaic *Homo* also exhibit higher frequencies of a protostylid. The frequencies of this feature in early modern humans and recent modern humans are within 20% (Table 1, SI-Table 10). Interestingly, the differences among hominins are not only regarding the frequencies but also in their form. In Late Pleistocene and contemporary modern humans, the protostylid is usually associated with the buccal groove. But in earlier hominins such as Asian *H. erectus* or the Dmanisi hominins, the protostylid is more cingulum-like, expressed as a cleft or badge that extends across most of the buccal surface of the molar^37^. This pattern of expression is even more pronounced at EDJ level^47,48^. As with the Carabelli’s trait, the protostylid of Dushan 1 is very pronounced and cleft-like involving nearly the whole upper buccal surface of protoconid and hypoconid. We consider that the morphology of the protostylid of Dushan1 represents a primitive feature shared with some Middle Pleistocene *Homo* in Eurasia.

#### Three-rooted P_3_

**Figure 6:**
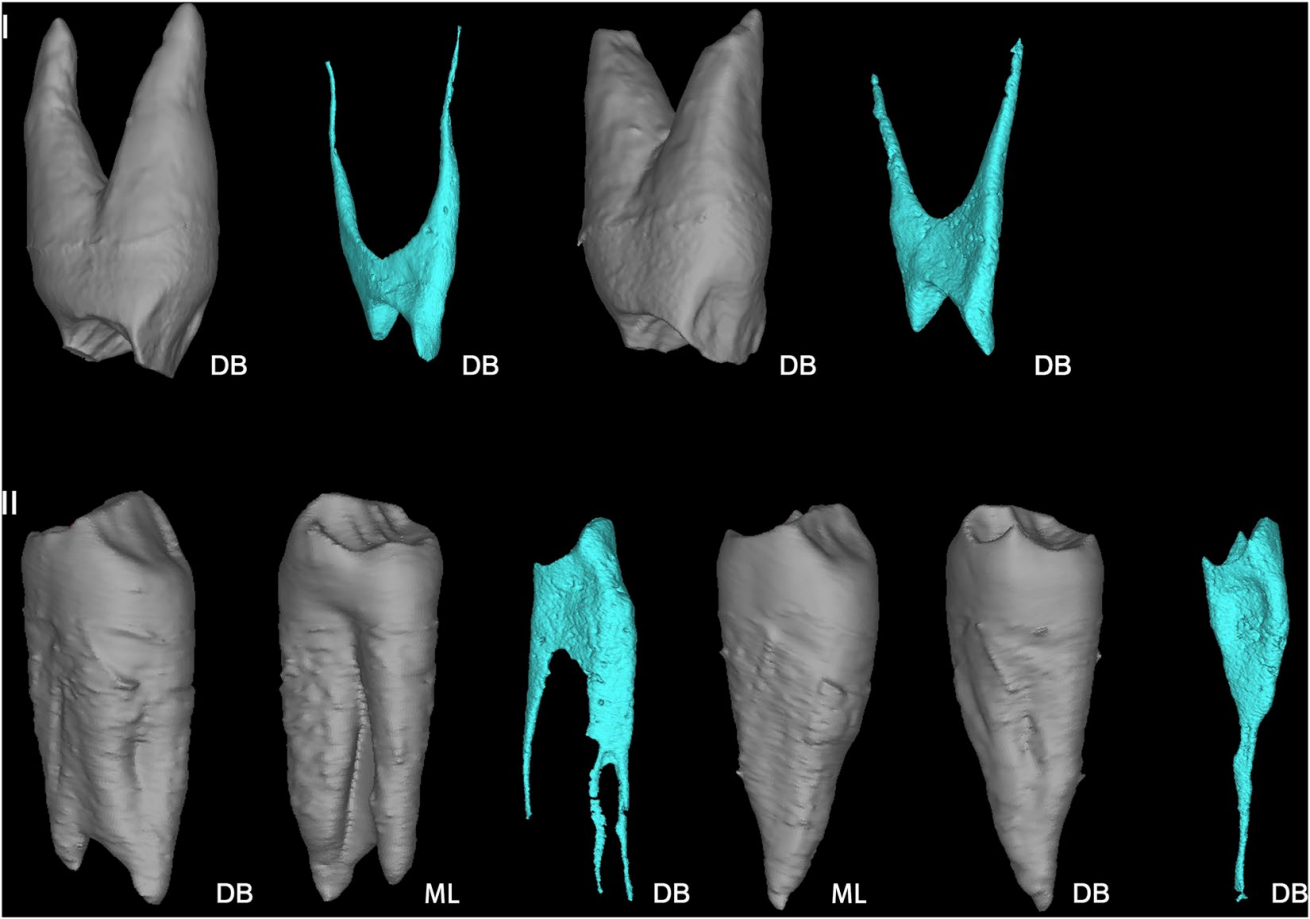
Roots and root canals of Dushan 1 premolars. I: right P^3^ and P^4^; II: right P_3_ and P_4_. DB: disto-buccal view; ML: mesio-lingual view (All the EDJ images are not scaled).

Previous studies indicate that upper and lower premolars tend to reduce their root number throughout the evolution of the genus *Homo*. Nearly all the *Australopithecus* species have two-rooted P_3_s. 55.6% of African early *Homo* (*Homo habilis*)^49^ and about 50% of *Homo erectus* specimens from Java have clearly bifurcated external roots of P_3_s^32^. So far, we have not identified any two-rooted P_3_s in the Chinese Middle Pleistocene *Homo* record although a few *Sinanthropus* P_3_s (nos. 21, 23, 82) from Zhoukoudian exhibit Tomes’ roots^50^. The P_3_s of European Early Pleistocene hominins are all two-rooted with either Tomes’ root (Sima del Elefante) or a clear bifurcation corresponding to a 2 R: MB + DL pattern (Gran Dolina-TD6: *H. antecessor*) following Wood’s standard (49 and authors’ observations of the ATD6-96 with micro-CT scanning). European Middle Pleistocene hominins and Neanderthals usually have single-rooted P_3_s^30,51,52^. The frequencies of two-rooted P_3_s in global modern humans are very low, ranging from 1.0% to 6.6% with average of 3.6%^53,54^. Three-rooted P_3_s are very rare in both fossil hominins and recent modern humans^27,30,32,33,54^. This feature was observed in some non-human primates like *Pan*, *Gorilla, Pongo* and *Hylobates* with frequencies ranging from 1–50%^32^. For fossil hominins, three-rooted P_3_s were only found in *Australopithecus africanus*, *Australopithecus afarensis* and *Paranthropus robustus*^32,55^. The three-rooted P^3^s in modern human populations are even rare with reported occurrence in global modern human populations as 0.2% (1/584)^54^. The micro-CT observation of both Dushan P_3_s reveal that they are three-rooted with a 3-C-1 form as defined by Moore (ref.^55^; Fig. 6). Thus, the three-rooted P_3_s observed in Dushan 1 represent the retention of another primitive feature in this specimen.

#### Two-rooted P^4^

As shown in Fig. 6, both P^3^s and P^4^s of Dushan 1 are two-rooted, with a pronounced bifurcation. Most upper premolars of Middle Pleistocene hominins are two rooted. For example, 64.3% and 87.5% of the Atapuerca SH P^3^s and P^4^s are two-rooted, respectively^30^. However, two-rooted P^4^s are very infrequent in Late Pleistocene and contemporary modern human groups, ranging from 0% to 4.3% in our Late Pleistocene and Chinese modern sample, respectively^33^. Therefore, a two-rooted P^4^ is an additional primitive trait in Dushan 1, more commonly found in Middle Pleistocene hominins.

#### Complex occlusal morphology at EDJ of molars

**Figure 7:**
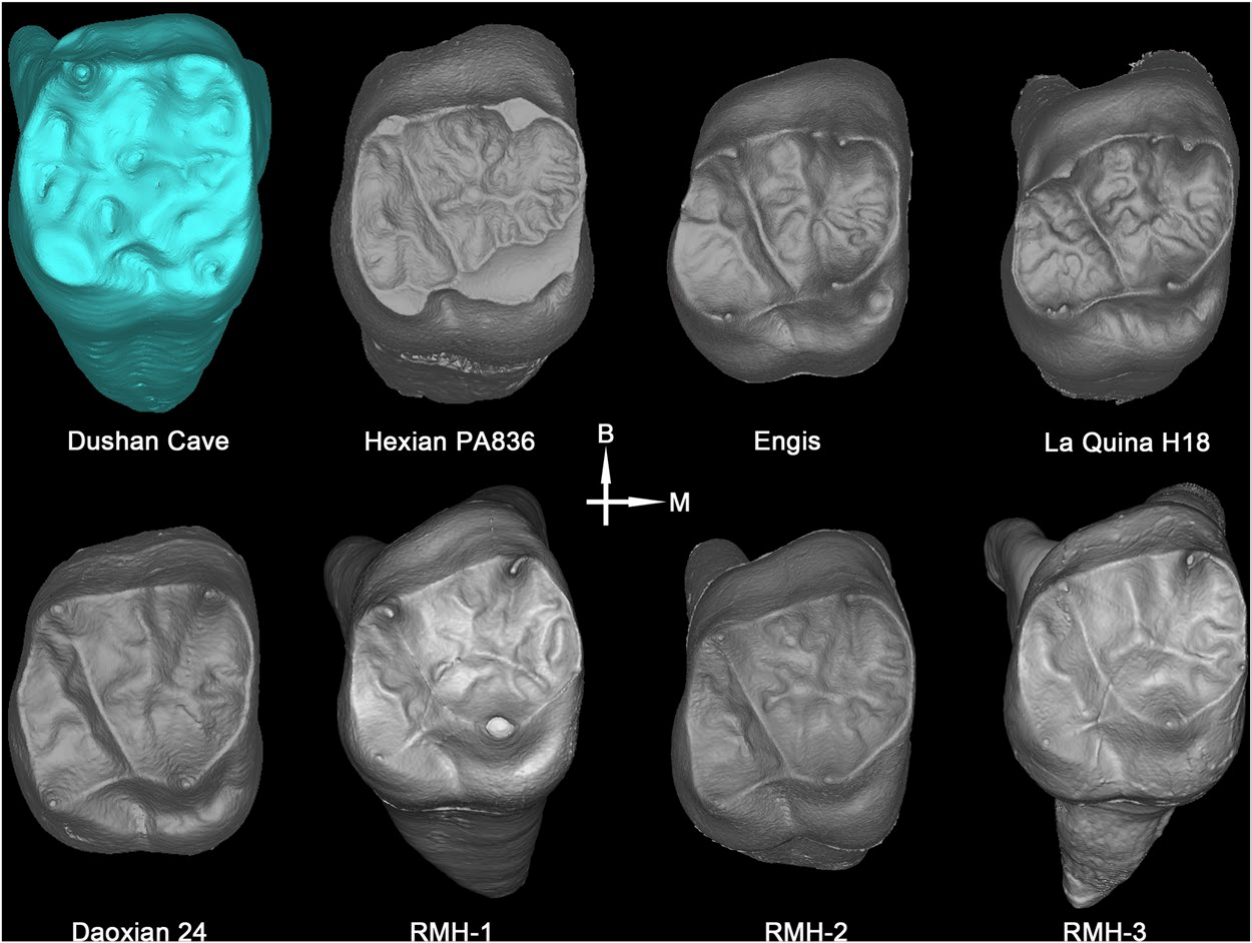
Comparison of M^1^ occlusal morphologies at EDJ between Dushan 1 and comparative samples.

Micro-CT scanning shows that the EDJ of all the post-canine teeth of Dushan 1 is characterized by complex patterns of ridges, furrows and crenulations. This is particularly pronounced in upper and lower molars (Fig. 7, SI-Fig. 15). Recent studies of East Asian Middle Pleistocene *Homo* populations (Xujiayao, Hexian, Yiyuan, Zhoukoudian) reveal that all these hominins exhibit complex EDJ morphologies in their post-canine teeth coined as “dendrite-like” EDJ^8,41,42^. This “dendrite-like” surface is absent in the Late Pleistocene early modern humans from East Asia such as Daoxian, Liujiang or Tianyuan Cave. In the present study, we analyzed the EDJ surface of 153 modern Chinese molars. Only 5.2% (8/153) showed a complex EDJ pattern and none reached the level of “dendrite-like” EDJ characteristic of the Asian *H. erectus* groups mentioned above (Table 1, SI-Table 7). In light of these comparisons, the complex EDJ surface morphologies of Dushan 1 molars should be considered a primitive character shared with Middle Pleistocene archaic *Homo*.

#### Buccal basal swelling

**Figure 8:**
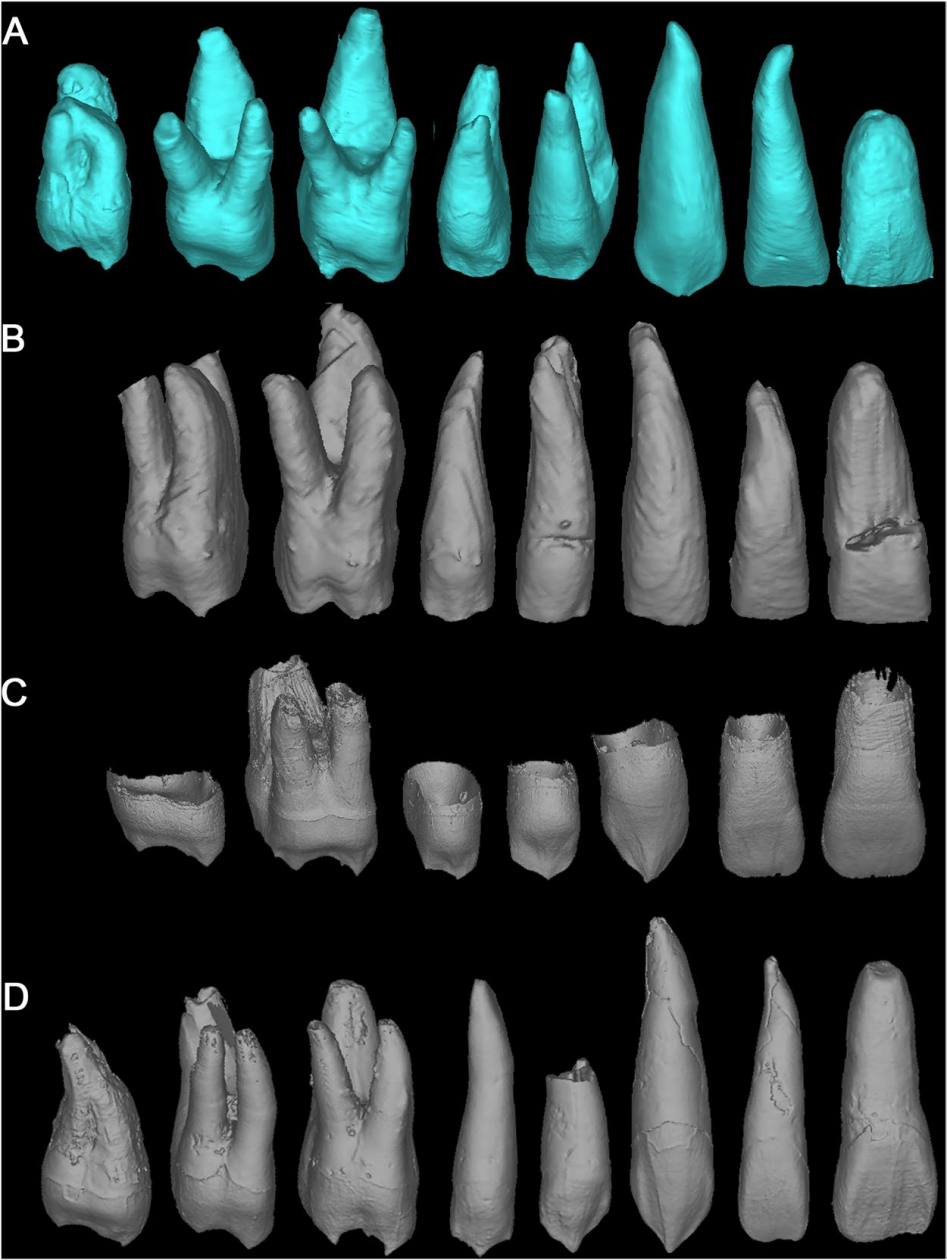
Buccal EDJ views of upper dentitions for Dushan 1 and comparative samples. (**A**) Dushan 1. (**B**) Liujiang. (**C**) Neanderthal (La Quina H18). (**E**) Modern human.

The expression of a strong basal bulging at the buccal surface or premolars and molars is more frequently observed in earlier hominins (*Australopithecus*, early *Homo* and Middle Pleistocene *Homo* specimens from Asia) and is usually interpreted as a cingular derivative^50,56–59^. One of the most remarkable features of the upper and lower premolars and molars of Dushan 1 is the expression of a strong basal swelling of the buccal surface at the dentine level that in the case of the premolars is continuous with the marked marginal ridges (Figs 3, 5 and 8) (see buccal vertical groove complex below) thereby forming a cingulum. The frequency of this feature in Middle Pleistocene *Homo* specimens from China is very high (50–90%) and decreases substantially in Late Pleistocene early modern humans (12.9–14.6%), with only more gracile expressions observed at the EDJ level. This feature is very rare in contemporary modern humans (less than 10%) and can be regarded as a primitive trait of Dushan 1 individual.

#### Crown buccal vertical groove complex

Previous studies showed that *Australopithecus* and *Homo habilis* premolars exhibit high frequencies of vertical grooves on the mesial and/or distal aspects of the buccal surfaces^56,59,60^. This feature was also identified in some Asian and European Early and Middle Pleistocene hominins with frequencies ranging from 12% to 80%^30,31,37,50^. In the present study, we found pronounced buccal grooves in the crown of all the upper and lower canines, and premolars of Dushan 1, and those were particularly pronounced at the EDJ surface. Similar grooves can also be seen in Dushan 1 lower molars although they constitute part of the protostylid. The premolars of some Chinese Middle Pleistocene *Homo erectus* exhibit a buccal vertical groove either at both the OES and EDJ (Hexian PA832 and HXUP, Zhoukoudian PA67, PA68, PA110) (ref.^41^, present study) or just at the EDJ (Yiyuan Sh.y. 003, Sh.y. 071)^42^. However, this feature is absent from nearly all the Late Pleistocene Chinese samples such as the Xujiayao, Liujiang and Tianyuan Cave sites. None of the recent modern human dentitions studied here revealed this feature. There is a Neanderthal specimen (La Quina H18) that shows a well demarcated essential ridge and relatively pronounced vertical grooves at the EDJ, but these do not reach the degree of expression seen in Dushan 1, and they lack the pronounced angle between the basal part of the crown and the relatively depressed buccal slope of the buccal cusps. The involvement of canine, premolars, and molars is also unusual (Figs 3, 5 and 8). We name this composite feature as “crown buccal vertical groove complex”, and its expression in the upper and lower dentition of Dushan 1 can be considered a primitive trait.

The result of the PCA based on eight non-metric traits for Dushan 1 and comparative samples show that Dushan 1 stays closer to the East Asian archaic *Homo* and early modern humans than to the recent modern humans with 79% variation represented by the first two PCs (see SI-5 and SI-Fig. 8 for detail).

## Discussion

In the present study, we comprehensively analyzed the dental morphology of Dushan 1 individual, a terminal Pleistocene hominin from Southern China, by combining various metric and non-metric characters. Through comparisons with Middle Pleistocene *Homo* populations, Late Pleistocene early modern humans and recent modern humans as well as some reference to the variability of African early hominins, the morphological status of each dental character of Dushan 1 was determined. Although possible inter-character correlations and the exact taxonomic significance of each trait are not fully understood, our comparisons are based on large samples of Pleistocene *Homo* and early and recent modern humans that can help us interpret the dental pattern of Dushan 1 more reliably. As shown in Table 1, 15 out of 25 key traits in Dushan 1 are in common in either Eurasian Pleistocene archaic *Homo* (13 traits) or African early hominins (4 traits). Of those, only 10 traits are shared with modern humans.

The mosaic combination of *H. sapiens* trait with an unusual number of primitive features and a few unique features makes Dushan 1 an “atypical *H. sapiens*” and raises questions about the evolutionary meaning of such a combination of dental characteristics in a terminal Pleistocene in Southern China. Among the “atypical” dental traits of Dushan 1, we highlight: (1) an overall large dental size; (2) unusual dental size proportions that fall outside the range of variation of modern humans, including recent Chinese populations; (3) pronounced cingulum-like Carabelli’s trait and protostylid in molars; (4) the pronounced marginal ridges, and buccal vertical grooves on upper and lower premolars that merge with a basal bulge forming a cingulum-like structure; (5) the unusual high combination of ancestral features such as three-rooted P_3_, strongly and divergent bifurcated essential ridges of upper premolars, accessory marginal tubercles, strong development with conspicuous tip of the C5, expression of C6 and C7 (see SI-4);(5); the “dendrite-like” occlusal EDJ surface morphology in both upper and lower M1 and M2; (6) the rare “crown buccal vertical groove complex” from the canines to the third molars of both upper and lower dentitions.

Due to the high variability that is characteristic of *H. sapiens*, some of these primitive or uncommon traits may not be taxonomically diagnostic per se, being occasionally present, albeit in very low percentages, in some recent modern humans^27,28,30,33^. Nevertheless, the combined expression of at least 13 characters listed in Table 1 (nos #3, #13, #14, #21, #22, #24, #27, #39, #49, #62, #63, #68, #69) for which Dushan 1 exhibits pronounced expression are more frequent and have more pronounced forms in archaic *Homo* and can easily differentiate earlier hominins from modern human groups.

In addition, the primitive dental traits of Dushan 1, such as the three-rooted P_3_ and the vertical grooves on the premolar crown buccal surface, are widely present in different Middle and Late Pleistocene hominins (Chinese *H. erectus*, European Middle Pleistocene hominins, Javanese *H. erectus*, and other later archaic *Homo* such as Xujiayao) or even more frequent in *Australopithecus* and early *Homo* specimens.

Although the exact ontogenetic mechanism for multirooted P_3_s is not fully understood, and it seems to be controlled by either genetic or epigenetic factors, the available data indicate that multirooted mandibular premolars are early-evolved, highly conserved independent phenotypes that extends back to early primates. High occurrences of mandibular premolar multirooted variants are also observed in in our megadont hominin ancestors^54^. With all these in mind, the three-rooted P_3_s of Dushan 1 more possibly reflect a primitive retention.

In the later Pleistocene *Homo* species, the frequencies of the vertical grooves on the premolar crown buccal side decreased by a 30–40%. In Dushan individual, the grooves are present in upper and lower canines, premolars and molars and particularly pronounced at the EDJ surface, whereas none of our comparative samples of hominin fossil and recent modern humans with whole dentitions exhibits this composite character.

Summarizing, our analyses reveal that Dushan 1 exhibits a special combination of primitive and unusual dental traits on the post-canine dentition together with some *H*. *sapiens* features.

For the past ten years, several studies point to Southwest China and adjacent Southeast Asia as a key place to study the variability of Late Pleistocene hominins, including our own species^11,12,19,20,24,25^. The analysis of Late Pleistocene samples like those from Maludong and Longlin have been interpreted as evidence of complex demographic patterns in Southwest China with several migrations and possible coexistence of *H. sapiens* with archaic hominins^21–23^. In this context, finding a suite of primitive traits and a few unusual features in Dushan 1 provides additional information and fossil evidence for the variability and diversity of terminal Pleistocene human evolution in this region. There are a number of alternative explanations for the mosaic dental pattern of Dushan 1.

One alternative is that the primitive features expressed by Dushan are the result of introgressive hybridization with a late persisting archaic hominin. As mentioned above (Table 1), most of the primitive features identified in Dushan 1 are more often found in Middle and early Late Pleistocene archaic *Homo*. Some special features, such as shelf-like Carabelli’s trait, are also found in the Late Pleistocene archaic hominins from Xujiayao. In addition, a series of mitochondrial and nuclear DNA analysis of a finger phalanx and two molars from the Denisovan remains revealed that the Denisovan genome has contributed 5% of the DNA to the genomes of present-day people in Oceania^13,14,16^. Recent studies show that the Late Pleistocene Xujiayao teeth share similarities to those of Denisovans including cingular remnant-like Carabelli’s cusps, strongly divergent roots and relatively large and complex crowns (refs^8,45^). All these studies suggest possible genetic contributions of late archaic hominins like Xujiayao and Denisovans to the ancestors of present-day populations across Asia and Oceania in addition to the Altai Mountains. The Denisovans may have lived in other parts of Asia^61–64^. The relatively high level of Denisovan introgression in present-day Southeast Asian populations suggest that Denisovans had a wide geographical range, possibly including South China. In this context, the suite of primitive traits identified in Dushan 1 could be due to introgression from some late archaic hominins such as Denisovans into Dushan ancestors. However, the limited morphological data available for these late archaic hominins, including Denisovans, as well as the lack of genetic data from Dushan 1 prevents a proper testing of these hypotheses.

A second alternative is that Dushan 1 represents one of the earliest *H. sapiens* populations inhabiting this region and, hence, the higher number of primitive traits retained by this group in comparison to later migrations. Recent studies of cranial, mandibular and dental morphologies of Middle and Late Pleistocene humans in Asia reveal high levels of variability. The combination of primitive and derived features does not follow a temporal gradient, and samples that are roughly contemporaneous may show a different combination of traits^7–10,41,42^. Based on these findings, it was suggested that such a mosaic pattern reflects an intermittent and/or discontinuous settlement of the continent. Fragmentation and isolation of the populations would have favored the retention of primitive features in certain groups and the development of new and/or more derived features in others^65,66^. The present study shows that except for a group of primitive features, Dushan 1 upper and lower dentitions also exhibit some very rare or unique morphological features, including three-rooted P_3_ and a “crown buccal vertical groove complex”. Considering these, alternatively, we propose that Dushan 1 may represent a late survival of one of the earliest modern human groups to settle in an isolated region of Southern China and thus, that the unusual high retention of primitive traits in this population would be indicative of its early origin. In this case, Dushan 1 would be representing a particularity of the populations inhabiting this region.

## Materials and Methods

### Materials

Except the left M^3^, the whole upper and lower dentitions of Dushan 1 are available for study. Some teeth are attached to their corresponding maxillary or mandibular bone (left C^1^ to M^2^, right M^1^ to M^3^, right I_2_ to M_1_, and right M_3_) (Fig. 1, SI-Fig. 3) whereas the rest are isolated (see SI-2 for details).

Our study focuses on the comparison of Dushan 1 individual with fossil hominins and recent modern humans in the region. Considering its terminal Late Pleistocene age and its possible contribution to the understanding of the complex evolutionary scenario of East Asia in this period, Dushan 1 was compared with East Asian Middle Pleistocene archaic *Homo*, Late Pleistocene early modern humans and contemporary populations to assess its biological affinities with them. Several recent modern human populations from archaeological collections in China are included, and some metric and non-metric data of global *Homo sapiens* samples from the literature are also used. The specimens of East Asian Middle Pleistocene archaic *Homo* comprised Chinese *Homo erectus*, and later archaic *Homo* members that were traditionally called “archaic *Homo sapiens*”. These two groups have been pooled together to provide a broad frame of reference for the expression of primitive traits in continental East Asia and to differentiate them from *Homo sapiens sensu stricto* (fossil and recent). To provide a broader view of Asian *H. erectus*, data from Javan *Homo erectus* are also included. Given their clear morphological differences but also their contemporaneity with *H. sapiens*, the metric and non-metric data of Neanderthals were also included. Finally, when necessary, the expression of the relevant traits in *Australopithecus*, African Early and Middle Pleistocene *Homo* (*H. habilis* and *H. ergaster*) and the European Early and Middle Pleistocene hominins was mentioned. Detailed information of the composition of the comparative samples is provided in SI-3 and SI-Table 3.

The EDJ and root morphology of Dushan 1 teeth were compared against a sample of Late Pleistocene and recent individuals preserving complete upper and lower dentitions (see SI-Table 4 for details).

### Methods

Dushan 1 dentition was studied through the following analytical approaches:

#### Traditional metric analyses

Mesiodistal (MD) and buccolingual (BL) dimensions of the crown were measured using a standard sliding caliper and recorded to the nearest 0.1 mm following^34^. Root length was measured from cervical line to the root tip on the buccal side. For the teeth embedded in the maxillary or mandibular bones, the measurement was made based on CT. With these metric data, crown sizes (MD and BL) of all the tooth types for both upper and lower dentitions were compared between Dushan 1 and comparative samples from East Asia. In order to compare dental size proportions, the crown size proportion between anterior teeth (I1 to C) and posterior teeth (P3 to M3), and molar size increase (%) were calculated^18,31^. To further help interpret the crown size proportion of Dushan 1, we compared the BL widths of the upper and lower I2, P3, and M1 of Dushan 1 against those of several modern and fossil specimens including Neanderthals, and Late Pleistocene and recent modern human populations from China, the Levant, North Africa and the Canary Islands (SI-Table 4). We ran three sets of analyses: one with all specimens that included the full set of maxillary dental measurements, one with all specimens that included the full set of mandibular dental measurements, and one with all specimens that included the full set of both maxillary and mandibular dental measurements. In each case, we derived size-corrected metrics in the form of residual values from linear regressions of log-transformed BL width against the log-transformed geometric mean of all relevant dental measurements (maxillary, mandibular, or maxillary and mandibular). With each set, we ran a Principal Component Analysis (PCA) to visualize the shape of Dushan 1 in the context of shape variation in the entire sample. All analyses were conducted in R version 3.4.0^67^. The results of this analysis are shown in SI-Figs 4–6.

#### Non-metric trait comparison

Our study assessed both the external morphology of the original Dushan 1 teeth and the virtual reconstruction from micro-CT scanning of the EDJ and the root. Tooth wear stages were scored following the standards set by Molnar^68^. The terms employed for the non-metric description were taken from references^27,37,49–51,60,69^. Some of the non-metric features were scored using the Arizona State University Dental Anthropology System (ASUDAS)^27^. Apart from the ASUDAS traits, in this study we have defined and recorded other traits we consider as relevant for the characterization of Dushan 1.

To further explore the composite morphological information of the non-traits, we conduct PCA based on eight non-metric traits of I^1^ labial convexity, P^3^ bifurcated essential crest, P^3^ mesial and distal accessory ridge, P^3^ accessory marginal tubercle, M^1^ Carabelli’s trait, M_1_ protostylid, P^3^ crown buccal vertical groove and complicated occlusal morphology at EDJ for molars. The result of the analysis is provided in SI-5 and SI-Fig. 8.

#### Crown buccal groove of upper and lower postcanine dentitions

Previous studies showed that premolars of *Australopithecus* and early *Homo* frequently exhibit vertical grooves on the crown buccal side^56,59,60^. These grooves delimit thickened marginal ridges that are commonly merged with a pronounced basal bulge or band-like prominence at the buccal surface. Our observation of Dushan 1 maxillary and mandibular dentitions indicates that the vertical grooves are present in all the post-canine teeth of this individual. Therefore, this trait has been also recorded in our comparative samples (SI-Table 5). Following the approach by Wood and Uytterschaut^60^ and Kaifu^31^, either if one or both of the buccal grooves are expressed we record the trait as present.

#### Micro-CT scanning and reconstructions of the enamel dentine junction (EDJ) surface and roots

All Dushan 1 teeth and some specimens of comparative samples were scanned by high resolution microcomputed tomography (micro-CT) in order to virtually reconstruct the enamel-dentine junction (EDJ) and roots which are embed in the jaw bones. A 225 kV-µCT scanner was used to scan the specimens under the following settings: 140 kV, 120uA, 0.5 angular increment one step, 360 degrees of rotation, and the average of 4 frames. The isometric voxel size is 36.1 µm. A total of 720 transmission images were reconstructed in a 2,048*2,048 matrix of 1,195 slices. The RAW volume was imported into Mimics 19.0 to complete the virtual reconstruction.

All the key traits observed, measured and compared in the present study are listed in SI-Table 10. In addition, detailed morphological descriptions and comparisons for each individual tooth are also conducted (see SI-4).

## Supplementary information

SI for Dushan Cave teeth

## Data Availability

All data generated or analyzed during this study are included in this published article (and its Supplementary Information Files).
