# Supplementary material for: Mosaic dental morphology in a terminal Pleistocene hominin from Dushan Cave in southern China
Source: Sci Rep. 2019 Feb 20;9:2347. doi: 10.1038/s41598-019-38818-x (PMC6382942; doi:10.1038/s41598-019-38818-x)
Supplement: Supplementary file 1 — SI for Dushan Cave teeth [file 41598_2019_38818_MOESM1_ESM.docx]

**Mosaic dental morphology in a terminal Pleistocene hominin from Dushan Cave in southern China-Supplementary Information**

Wei Liao^a,b^, Song Xing^c,d^, Dawei Li^a,b^, María Martinón-Torres^e,f^, Xiujie Wu^c,d^, Christophe Soligo^e^, José María Bermúdez de Castro^e,f^, Wei Wang^g*^, Wu Liu^c,d*^

a State Key Laboratory of Geological Processes and Mineral Resources, School of Earth Sciences,

China University of Geosciences, Wuhan 430074, China

b Anthropology Museum of Guangxi, Nanning 530028, Guangxi, China

c Key Laboratory of Vertebrate Evolution and Human Origins of Chinese Academy of Sciences, Institute of Vertebrate Paleontology and Paleoanthropology, Chinese Academy of Sciences, Beijing 100044, China

d CAS Center for Excellence in Life and Paleoenvironment, Beijing, 100044, China

e Department of Anthropology, University College London (UCL), 14 Taviton Street, London WC1H 0BW, UK

f National Research Center on Human Evolution (CENIEH), Paseo Sierra de Atapuerca s/n. Burgos 09002, Spain

g Institute of Cultural Heritage, Shandong University, 72 Jimo-Binhai Road, Qingdao 266237, China

^*^Corresponding authors.

Wu Liu (Address: No. 142 Xiwai Street, Beijing 100044, China; E-mail: [liuwu@ivpp.ac.cn](mailto:liuwu@ivpp.ac.cn))

Wei Wang (Address: No. 72 Jimo-Binhai Road, Qingdao 266237, China; E-mail: [wangw@sdu.edu.cn](mailto:wangw@sdu.edu.cn))

**SI-1 Dushan Cave site and dating**

**SI-2 Dental remains of Dushan 1**

**SI-3 Comparative samples**

**SI-4 Metric analysis**

**SI-5 Morphological descriptions of individual tooth**

**SI-6 Figures and Tables**

**SI-7 References**

**SI-1 Dushan Cave site and dating**

Dushan Cave is located in Linfeng Town, Tiandong County, Guangxi Zhuang Autonomous Region in South China (23°30'8.0"N, 107°8'5.5"E, SI-Fig. 1).The site was investigated and excavated in 2011 by the authors (DWL and WW) and their colleagues from the Natural History Museum of Guangxi Zhuang Autonomous Region.


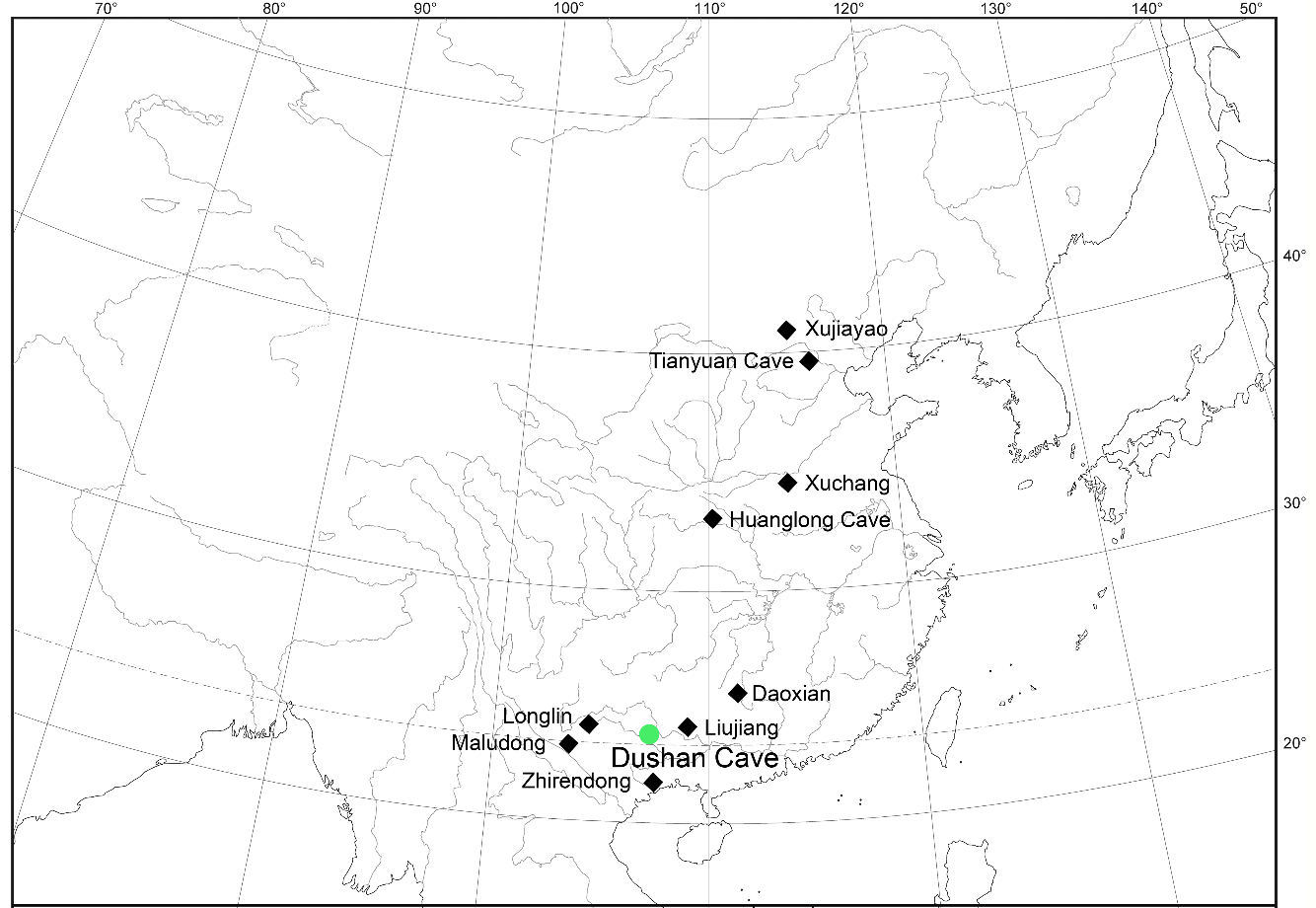


SI-Fig. 1. Geographic location of Dushan Cave and some Late Pleistocene hominin fossil sites in China.

Dushan Cave has one main chamber (30 m long) with a northwest-facing entrance that is the widest part (9 m) of the cave. An area (of 3.15 m×1.8 m) near the middle part of the gallery was excavated to a maximum depth of 1.9 m (SI-Fig. 2A). The cave deposits are divided into four major units from top to bottom, as follows (SI-Fig. 2B):

Unit I is cemented yellow silty clay with the major distribution in the northeast corner of the excavation, containing a small number of stone artifacts and sparse teeth of large mammals (*Sus* sp. and *Cervus* sp.) and fragmentary bivalves and gastropods.

Unit II is grey yellow silty clay containing large limestone breccias with the length up to ~ 20 cm. While chipped stone tools prevailed, polished stone implements began to appear in the base of the unit. In addition, we found a pottery fragment in the upper part of the unit. Some mammal teeth (*Sus* sp., *Cervus* sp. and *Hystrix* sp.) and fragmentary bivalves and gastropods were present as well.

Unit III is pale yellow silty clay with the major distribution in the southeast corner of the excavation, containing some gray weathered limestone breccia. All the human fossils including a cranium, the mandibular fragments, and the isolated teeth were found within a small area in unit III. In comparison with UnitⅡ, all the stone artifacts preserved in this unit are chipped stone tools without any polished stone implement. The first occurrence of the chipped artifacts is at a depth of 185 cm in the southeast corner of the excavation (SI-Fig. 2C). Like in Unit II, some mammal teeth (*Macaca* sp. *Sus* sp., *Cervus* sp. and *Hystrix* sp.) and fragmentary bivalves and gastropods were also present.

Unit IV is well cemented and hard pale yellow calcareous clay. This unit is restricted to the northeast corner of the excavation with no artifact and human fossil.

**Dating:** All the human fossils of Dushan 1 were found in the southeast corner of the excavation at the depth of 125 cm (SI-Fig. 2C). During the excavation, three charcoal samples were collected, one from Unit II at a depth of 85 cm and two samples from Unit III at depths of 145 and 165 cm, respectively. Accelerator mass spectrometry (AMS) radiocarbon dating tests were conducted on these samples at the Dating Lab of the School of Archaeology and Museology of Peking University. The charcoal sample from Unit II provided an age of 7753±49 years BP (SI-Table 1), precisely in the Neolithic period, and in line with the occurrence of polished stone implements found in this unit. The other two samples, from Unit III yielded ages of 14309±251 and 14995±369 years BP. Assuming that the accumulation rate of Unit III is roughly constant, chipped stone tools appeared at this site as early as ~15850 BP and Unit III may have continued up to ~12765 BP. It can reasonably inferred that the hominin fossils at Dushan Cave date to the period from ~15850 to ~12765 BP.


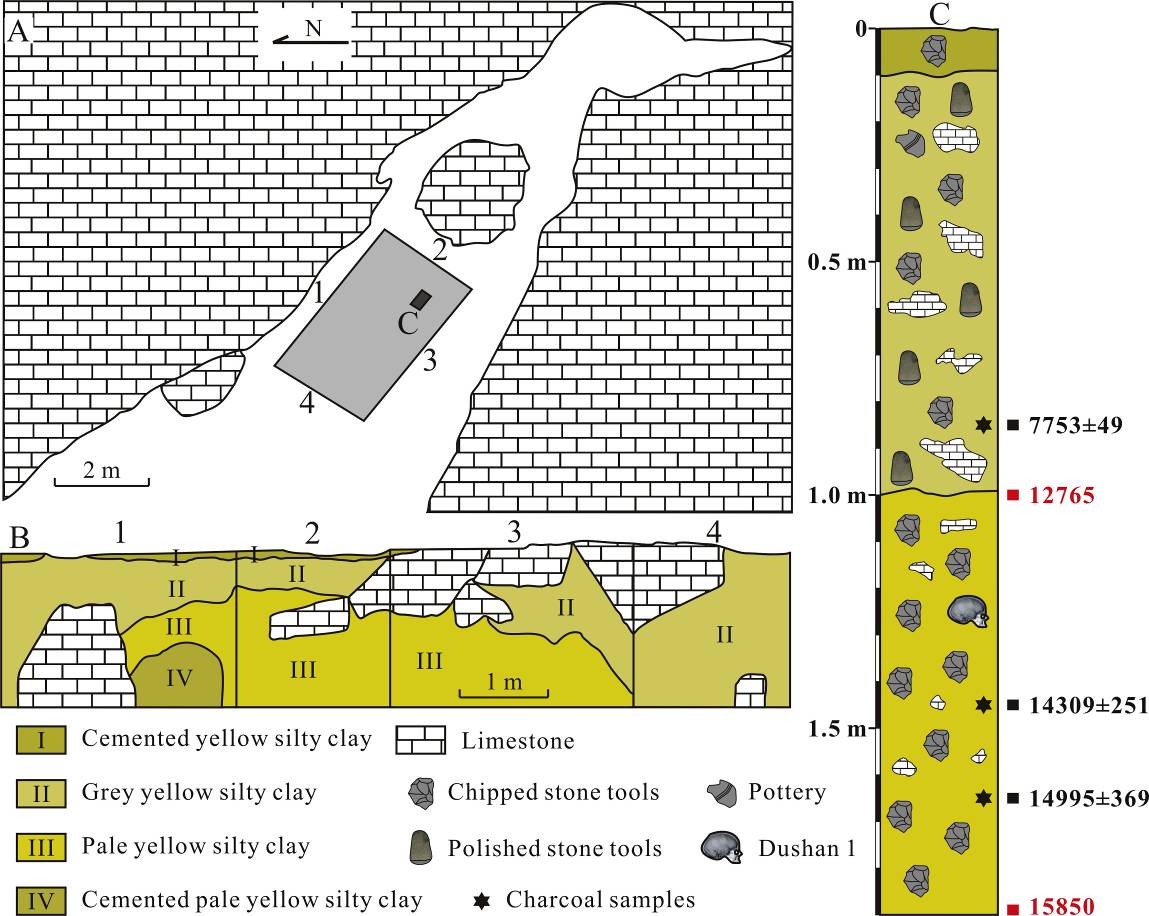


SI-Fig. 2. Site of Dushan Cave, Guangxi Zhuang Autonomous Region, South China. A, Plan of Dushan Cave with the excavation trench (gray rectangle). B, Cross-sections of the excavation trench. C, Stratigraphic profile of the southeast corner of the excavation trench (black rectangle in A). The presumed ages for Unit III (15850 and 12765 YBP) are in red according to the accumulation rate and two AMS ^14^C data.

SI-Table 1. Accelerator mass spectrometry ^14^C dating of charcoal samples from Dushan Cave, South China

| Sample no. | Lab no. | Layer | ^14^C BP Age | cal BP Age |
| --- | --- | --- | --- | --- |
| DSD-9 | BA10205 | II | 6915 ± 45 | 7753 ± 49 |
| DSD-15 | BA10206 | III | 12245 ± 45 | 14309 ± 251 |
| DSD-17 | BA10207 | III | 12640 ± 150 | 14995 ± 369 |

Calendrical years were calculated by using CalPal Online, version 1.5

**SI-2 Dental remains of Dushan 1**

The hominin fossils found at Dushan Cave comprise a nearly complete right calotte, most of the facial bones, some mandibular fragments, and the complete upper and lower dentitions except for the left M^3^ (left M^3^ was missing). All the cranial bone fragments fit well with each other and there is no duplication of any anatomical element. Therefore, it is logical to assume that all the cranial bones and teeth found at Dushan Cave come from the same individual, labelled as Dushan 1.


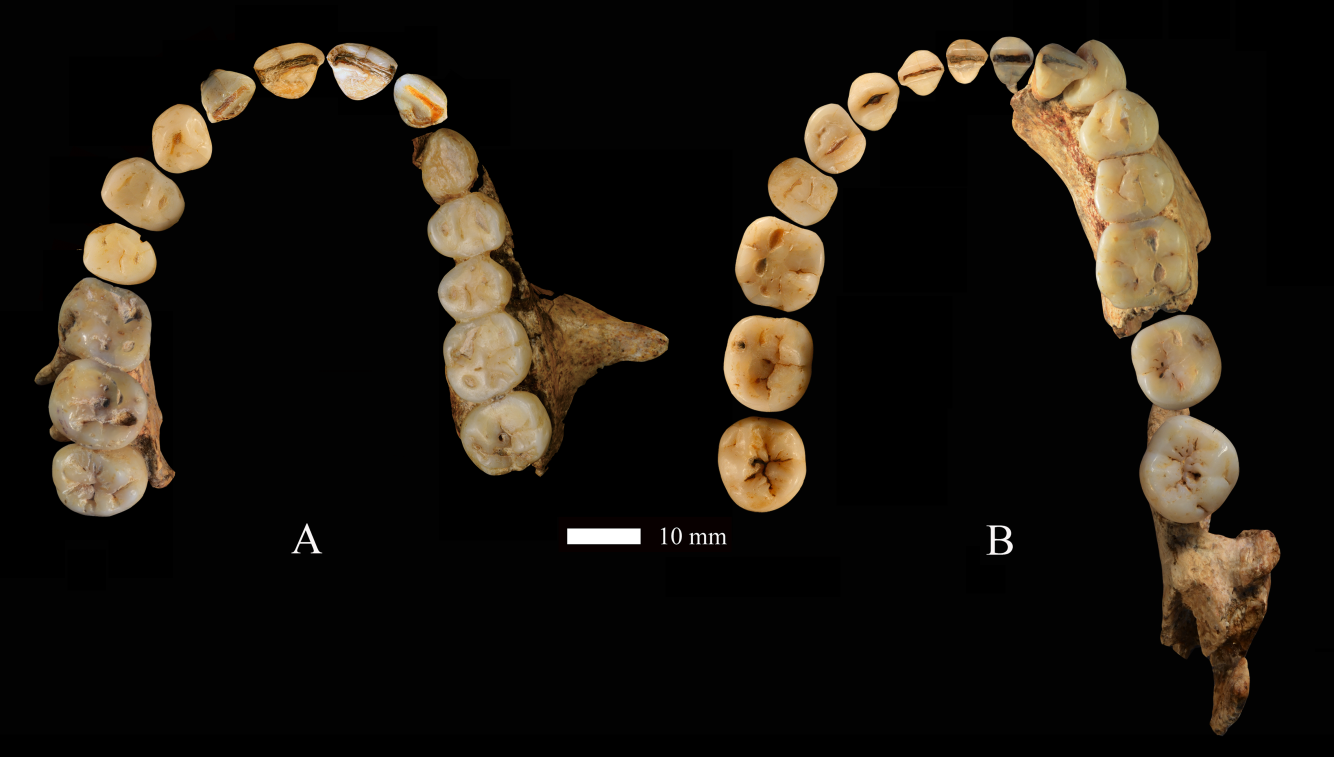


SI-Fig 3. The maxillary and mandibular dentitions of Dushan 1. (A: Maxillary dentition; B: mandibular dentition. The teeth are approximately placed with normal anatomical position)

The teeth of Dushan 1 from the right P^4^ to the left I^2^, and from the right I_1_ to the left M_3_ teeth were found isolated. The remaining teeth were found in situ in their corresponding maxillary and mandibular fragments (Fig. 1 and SI-Fig. 3). The present study will focus on the morphological and metric description and comparisons of the maxillary and mandibular dentitions of Dushan 1. Detailed information of each tooth, including crown and root measurements, is provided in SI-Table 2.

**Age and sex:** The cranial sutures of Dushan 1 specimen are visible. The preserved sagittal and coronal sutures are almost fused, whereas the lamboid, the parietal-mastoid and occipital-mastoid sutures are still clear. All these features point to a young adult. All teeth present moderate dental wear, ranging from grade 3 to 4 of Molnar’s standard (68), except the M^3^s. Emergence of the right M^3^ was incomplete. No tooth wear is apparent on the right M^3^ whereas the left M_2_ exhibits two minimal interproximal distal wear facets placed in a lower position. Therefore, the M^3^s were not functional. Given the degree of dental wear of the rest of the teeth, in normal conditions we would expect the M^3^ also presenting a higher degree of wear. This might indicate that something was preventing the proper eruption of the third molars. Considering some signs of dental crowding in Dushan 1, such as the lingual displacement of the right I_2_ against the lingual surface of the C, we hypothesize that the M_3_s may have not erupted properly because of space constraints in the arcade.

The reconstructed cranium of Dushan 1 indicates a robust individual. This, together with the expression of distal accessory ridges in upper and lower canines (see below) is suggestive of a young adult male.

SI-Table 2. Inventory and measurements of Dushan 1 dentitions

| Teeth | Side | Wear | Crown Diameters | | Cervical diameters | | Root length |
| --- | --- | --- | --- | --- | --- | --- | --- |
|  |  |  | MD | BL | MD | BL |  |
| Maxilla | | | | | | | |
| I^1^ | L | 5 | 9.1 | 7.2 | 7.7 | 7.1 | - |
| I^1^ | R | 5 | 9.5 | 6.9 | 7.6 | 7.2 | - |
| I^2^ | L | 5 | 8.1 | 6.9 | 5.8 | 6.4 | 15.4 |
| I^2^ | R | 5 | 8.0 | 6.6 | 5.6 | 6.3 | 15.1 |
| C^1^ | L | 3 | 9.0 | 9.2 | 6.1 | 8.6 | 14.8 |
| C^1^ | R | 4 | 9.0 | 8.9 | 6.2 | 8.1 | 14.2 |
| P^3^ | L | 3 | 8.5 | 11.3 | 5.7 | 10.6 | 10.6 |
| P^3^ | R | 3 | 8.3 | 11.5 | 5.4 | 10.3 | 10.8 |
| P^4^ | L | 3 | 8.0 | 10.7 | 5.3 | 9.1 | 12.1 |
| P^4^ | R | 3 | 8.0 | 10.8 | 5.4 | 9.7 | 9.0 |
| M^1^ | L | 4 | 12.8 | 12.7 | 8.5 | 12.2 | 9.4 |
| M^1^ | R | 5 | 12.4 | 11.4 | 8.3 | 12.0 | 10.0 |
| M^2^ | L | 3 | 11.0 | 13.0 | 8.4 | 12.4 | 9.6 |
| M^2^ | R | 4 | 13.2 | 13.5 | 8.1 | 11.1 | 9.7 |
| M^3^ | R | 3 | 10.7 | 13.2 | 7.4 | 10.5 | 6.5 |
| Mandible | | | | | | | |
| I_1_ | L | 4 | 5.9 | 6.4 | 3.6 | 5.9 | 14.3 |
| I_1_ | R | 4 | 5.8 | 6.2 | 3.3 | 5.6 | 13.3 |
| I_2_ | L | 4 | 6.5 | 6.8 | 4.1 | 6.1 | - |
| I_2_ | R | 4 | 6.8 | 6.8 | 4.1 | 6.6 | 12.2 |
| C_1_ | L | 3 | 7.8 | 8.2 | 5.2 | 8.3 | 11.3 |
| C_1_ | R | 4 | 8.0 | 8.5 | 5.0 | 8.1 | 15.7 |
| P_3_ | L | 4 | 8.4 | 9.5 | 6.0 | 8.2 | 9.0 |
| P_3_ | R | 4 | 8.2 | 10.5 | 6.4 | 9.8 | 13.0 |
| P_4_ | L | 3 | 8.3 | 10.0 | 5.5 | 8.1 | 10.3 |
| P_4_ | R | 3 | 8.4 | 10.5 | 5.8 | 9.5 | 12.1 |
| M_1_ | L | 4 | 12.0 | 11.8 | 8.6 | 9.6 | 11.3 |
| M_1_ | R | 4 | 11.4 | 11.7 | 9.7 | 9.8 | 11.0 |
| M_2_ | L | 3 | 12.2 | 11.5 | 9.7 | 9.4 | 14.8 |
| M_2_ | R | 3 | 12.2 | 11.5 | 9.4 | 9.2 | (10.4) |
| M_3_ | L | 2 | 12.3 | 11.2 | 9.0 | 9.0 | (9.6) |
| M_3_ | R | 2 | 12.1 | 11.3 | 8.5 | 9.2 | 9.9 |

**SI-3 Comparative samples**

SI-Table 3. Comparative samples used in the morphological and metric comparisons

| Samples | Specimen sites | Data source and references |
| --- | --- | --- |
| East Asian Middle Pleistocene archaic *Homo* (EAH) | Hexian, Hualong Cave, Jianshi, Lantian Chenjiawo, Lantian Gongwangling, Luonan, Mehuidong, Nanzhao, Yiyuan, Yuanmou, Yunxian Meipu, Zhoukoudian, Changyang, Chaoxian, Dingcun, Panxian Dadong, Jinniushan, Xujiayao, Tongzi, Dingcun, Penghu-1 | Except the data for Luonan, Penghu 1 and some Zhoukoudian teeth, cited from references (50,70,71), the data of all other specimens were collected from the original fossils housed at IVPP, Guangxi Museum and Shandong Museum. |
| Early modern humans (EMH) | | |
| East Asia  (EMH-EA) | Bailian Cave, Baojiyan, Zhangwu, Chuandong, Daoxian, Duan, Fusui Nanshan Cave, Huanglong Cave, Huli Cave, Jiande, Jimuyan, Lipu, Liujiang, Longlin Longdong, Longtanshan, Luna Cave, Maomaodong, Tiandong, Tiyuan Cave, Tubo, Upper Cave, Xichou, Xintai, Zhaotong, Zhirendong,  Maludong, Longlin | The data of Daoxian, Fusui Nanshan Cave, Huanglong Cave, Huli Cave, Jiande, Jimuyan, Lipu, Luna Cave, Maomaodong, Tiandong, Tianyuan Cave, Tubo, Xintai, Zhirendong were collected from the original fossils housed at IVPP, Guangxi Museum, Fujian Museum and Shandong Museum. The data for the other specimens are from references (21, 72-77). |
|  |  |  |
| Africa and West Asia  (EMH-ASW) | Qafzeh (3, 3A, 4, 5, 6, 7, 8, 9, 10, 11, 12, 12A, B11-70, Qafzeh B11-72, B11-101, B11-UNN-1, Q79-C11), Skhul (1, 2, 3, 4, 5, 6, 7, 8, 9, 9A,10, 11, B11-71, B11-101), KRM 13400, KRM14696, KRM 13400, KRM16424, Mumba X, Mumba VII  Mumba VIII, KRM13400. KRM16424 | Contributed by Wolpoff and cited from references (78-81). |
| Europe  (EMH-EU) | Abri Pataud (1, 2, 4, 6, 26, 24), Brno (Zabovresky)3, Combe Capelle 1, Cro-Magnon (2, 4, 5), Dolní Vĕstonice (3, 13, 14, 15, 16，37 ), Fontechevade 2, Isturitz (4, Series 6-7-6B, Series 6-7-B-1, Series 6-7-B-UNN), Le Rois (R50-5, R50-45, R50-6, R51-29, R54, R51-10, R51-21A, R40, R50-35, R51-17, A-R48, R50-40), Le Rois (R5-10, R50-31),  Les Vachons 1, Mladec (1, 2A, 1903 MX 1, 1904 MX 1, 1904 MX 2, 5453, 1881-T2, 1904 Md 5, 1905 MND 5', 1903 Md 1), Pavlov (1, Pavlov 1-R, 3, 28),  Predmosti (1, 2, 3, 4, 5, 7, 9, 10, 14, 18, 20, 22, 24, 26, 27, 259, 476, 3070), Zlaty Kun 1 | Contributed by Wolpoff and cited from reference (81). |
| Neanderthal  (NEA) | Amud (1,5), Arcy Sur Cure (Typical Mousterian) (8, 9, 2416, 2931, H1, L1=#5, S2), Chateauneuf (Hauteroche-w2)2, Ehringsdorf (6, 7, 8, 1036), Genay (Core D'OR)1, Gibralter 1, Hortus (2, 3, 4, 5, 6, 7, 8, 10, 11, 14, 549, 550, 553, 766, 1262) , Krapina (6, 26, 35, 37, 39, 42, 43, 44, 50, 52, 58, 75, 80, 86, 90, 92, 94, 97, 99, 100, 103, 104, 105, 107, 109, 110, 113, 114, 121, 126, 130, 131, 144, 146, 180, 191, A/B, B/A ,D, E, Mx-C, Mx-E, Mx-G, Mx-H, Mx-I , MX-J, MX L, Mx-K, Mx-M, MX-O, MX-P Mx-Q, N/N, D/D, F/H), Kulna 1, La Chaise (8A, 9, 13, 14, 21, 38, 8, 8B, 9, 17, 18, 19, 20, 21), La Ferrassie 2, Le Moustier 1, La Quina (5, 9, 18, 20), Monsempron (1, 2, 3, 4), Ochoz (1, 2,1-R), Pech de l'aze 1, Petit Puymoyen (1, 2, 3, 4), Regourdou 1,Saccopastore (1, 2), Saint-Cesaire 1, Sakajia 1, Shanidar (1, 2, 3, 4, 6), Spy (1, 2, 2-R, 3), St Cesaire 1, Subalyuk 1, Tabun (1, 1-1, 2-1, 2-3, 3-2, 3-3,, 3-4, 351, B1, B4, EB), Vindija (201, 206, 226，229, 231, 259, 286, 287, 2008, 289, 290) | Contributed by Wolpoff |
| Recent modern humans  (RMH) |  |  |
| China (RMH-CH) | Xiawanggang of Henan(Neolithic), Hubei (Bronze age), Hubei (recent populations) (N=250) | IVPP  Hubei Provincial Institute of Archaeology |
| Prehistoric Southeast Asia | Java, Malaysia, Vietnam (N=104) | Cited from reference (31) |
| Australia/Melanesia | New Guinea, Australia/Tasmania, Aborigine (N=49) |  |
| Southeast Asia | Philippine Negrito (n=77) |  |
| Southeast Asia | China, Chukuci, Korea, Mongol, Yukagir (N=18) |  |
| Africa | Bushman, South Africa, East Africa, West Africa (N=163) |  |
| Indo/Europe | Indea, German, Hungary, Poland, Sweden (N=79) |  |

SI-Table 4. Comparative dental sample. Number and origin of specimens with upper only, lower only, and upper and lower dentition in each taxonomic/population unit.

|  | Upper only | Lower only | Upper and lower |
| --- | --- | --- | --- |
| Dmanisi | - | 1 | 1 |
| Early *Homo sapiens*^a^ | 3 | 2 | 4 |
| Galdar | 7 | 3 | 13 |
| Iberomaurusian^b^ | 3 | 9 | 4 |
| Jinniushan | 1 | - | - |
| Liujiang | 1 | - | - |
| Modern Chinese | 4 | 10 | 8 |
| Neanderthal^c^ | 3 | 11 | 4 |
| Předmostí | - | 1 | 6 |

^a^ Skhul, Qafzeh

^b^ Afalou, Tafforalt

^c^ Amud, Ehringsdorf, Genay, Hortus, Krapina, Le Moustier, Monsempron, Shanidar, Spy, St. Césaire, Subalyuk, Tabun

SI-Table 5. Individual specimens with whole dentitions used in morphological comparisons of EDJ by micro-CT

| Specimens | Dentitions | Data sources |
| --- | --- | --- |
| Dushan 1 | Both upper and lower dentitions | Present study |
| Liujiang | Upper dentition | IVPP |
| Xujiayao | Upper dentition (left I1, C, P3, P4, M1, M2) | IVPP |
| Tianyuan Cave | Lower dentition (right I2, C, P3, P4, M1, M2) | IVPP |
| Neanderthal (La Quina H18) | Upper dentition (right I1, I2, C, P3, P4, M1, M2) | <http://paleo.esrf.fr> |
| Recent modern humans (N=5) | Both upper and lower dentitions | IVPP |

**SI-4 Metric analysis**

In this section, the crown MD and BL dimensions of all the upper and lower tooth types of Dushan 1 and comparative samples of early modern humans, Neanderthals and recent modern humans from diverse geographical localities are analyzed by Principal Component Analysis (PCA). As shown in SI-Table 6, first and second PCs explain 39.3% and 13.3%, and 46 % and 24.3% of total variations for maxillary and mandibular dentitions respectively. Nearly all the premolar and molar size variables have higher loadings in both maxillary and mandibular dentitions in PC 1. As shown in SI-Fig. 4, Dushan 1 falls at the furthest right side of the PC1 axis, indicating the great size of the posterior teeth. In addition, the PCA analysis separates fossil hominins and recent modern humans. However, Dushan 1 falls outside the range of variation of recent and fossil modern humans, and is closer to East Asian archaic *Homo* and Neanderthals.


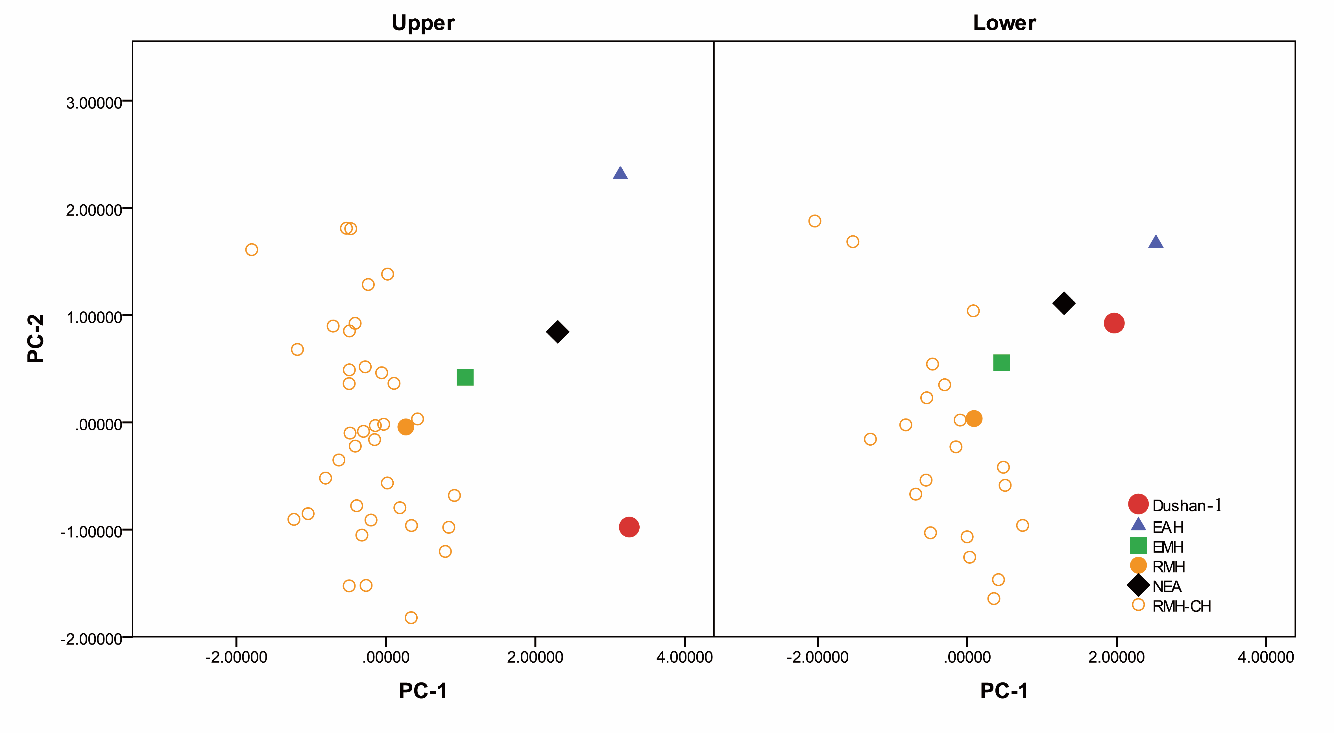


SI-Fig. 4. Results of the PCAs based on MD and BL crown diameters of upper and lower dentitions for Dushan 1 and comparative samples. EAH=East Asian archaic *Homo*; EMH=Global early modern humans; RMH= Global recent modern humans sample; NEA=Neanderthals; RMH-CH=Recent modern humans from China.

SI-Table 6. Component loadings and variance proportions of Dushan 1 individual teeth and the group-mean PCA crown diameters.

| Variables | Component loadings | | | |
| --- | --- | --- | --- | --- |
|  | Maxillary teeth | | Mandibular teeth | |
|  | PC-1 | PC-2 | PC-1 | PC-2 |
| I1 MD | .276 | .094 | .217 | .129 |
| I1 BL | .200 | .116 | .283 | .165 |
| I2 MD | .260 | .072 | .106 | .152 |
| I2 BL | .279 | .228 | .177 | .382 |
| C MD | .247 | .161 | .184 | .308 |
| C BL | .328 | .225 | .229 | .413 |
| P3 MD | .317 | .128 | .366 | .139 |
| P3 BL | .567 | .205 | .508 | .354 |
| P4 MD | .287 | .037 | .378 | .176 |
| P4 BL | .487 | .216 | .614 | .238 |
| M1 MD | .413 | .087 | .252 | .126 |
| M1 BL | .373 | .117 | .324 | .194 |
| M2 MD | .537 | -.160 | .555 | .239 |
| M2 BL | .503 | -.239 | .600 | .206 |
| M3 MD | .369 | -.410 | .834 | -.757 |
| M3 BL | .464 | -.517 | .812 | -.582 |
| Proportion of the variance (%) | 39.3 | 13.3 | 46.0 | 24.3 |
| Cumulative proportion (%) | 39.3 | 52.6 | 46.0 | 70.3 |

To further touch this issue, additional analysis with bivariate plots and PCA of the bucco-lingual widths of upper and lower I2, P3, and M1 of Dushan 1 and some specimens that preserve the three dental classes were conducted. As shown in SI-Fig. 5, bivariate plots of BL width against geometric means indicate substantial overlaps between samples with Dushan 1 characterized particularly by the relatively small size of its I^2^ but also, to a lesser extent, by the relatively small I_2_ and relatively large maxillary and mandibular P3s.


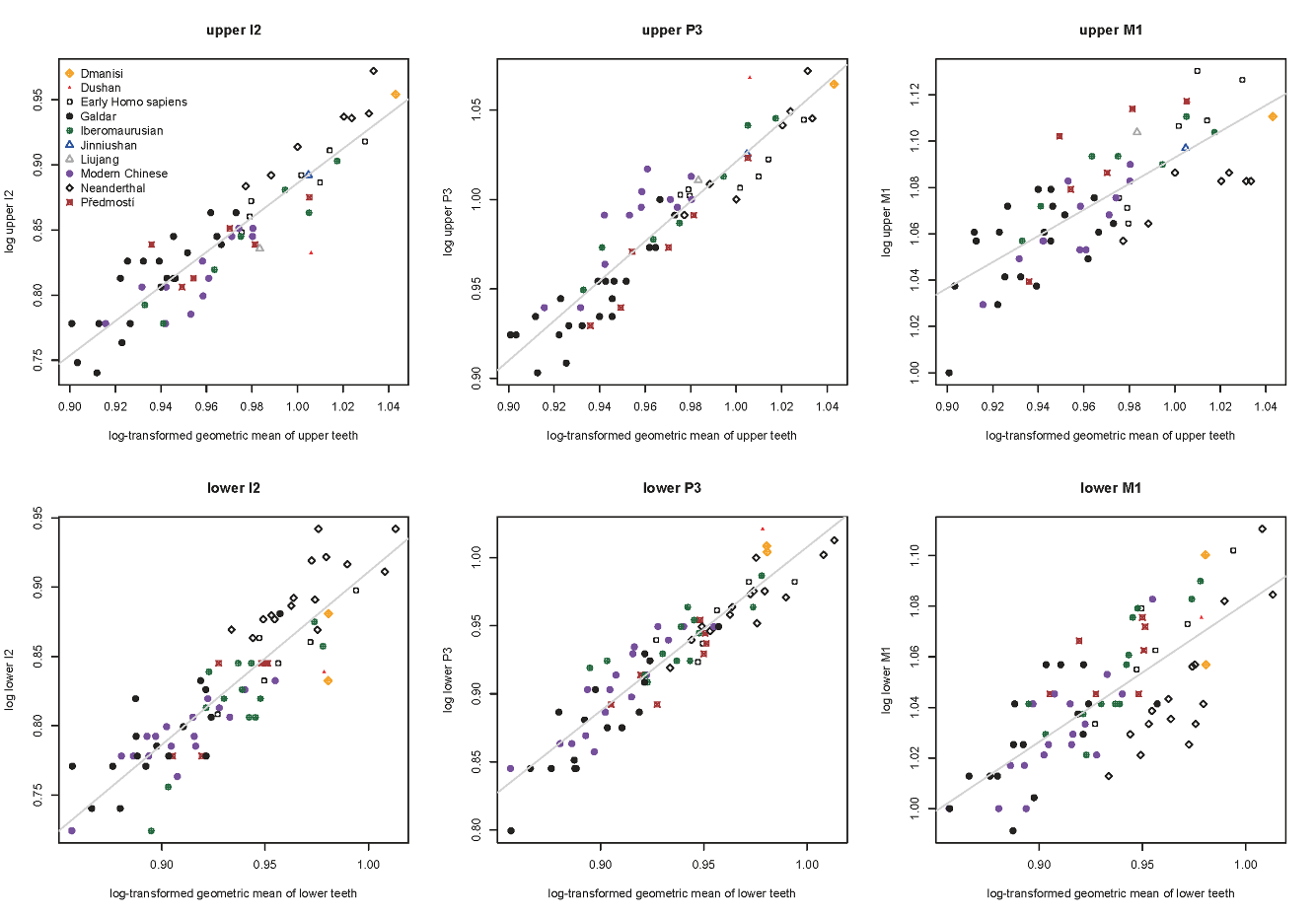


SI-Fig. 5. Scatterplots with linear regression line for each dental metric against the geometric mean of all dental metrics scored in the corresponding tooth row.

PCA of Dushan 1 and specimens from comparative samples (SI-Table 7, SI-Table 8, SI-Fig. 6, SI-Fig. 7) shows similar results. In combination, the metric analyses show that Dushan 1 has unusual dental proportions when compared to modern and fossil *Homo sapiens*, as well as to Neanderthals, and earlier *Homo* from Dmanisi. This pattern portrait Dushan 1 as an “outlier” or an atypical *H. sapiens*.

SI-Table 7. Principal component loadings for PCA of specimens for maxillary and mandibular teeth.

|  | Maxillary teeth | | Mandibular teeth | |
| --- | --- | --- | --- | --- |
|  | PC1 | PC2 | PC1 | PC2 |
| I2 residuals | 0.813 | -0.073 | 0.816 | -0.009 |
| P3 residuals | -0.343 | 0.741 | -0.400 | 0.712 |
| M1 residuals | -0.470 | -0.668 | -0.416 | -0.703 |

SI-Table 8. Principal component loadings for PCA of specimens preserving both maxillary and mandibular teeth

|  | PC1 | PC2 | PC3 | PC4 | PC5 |
| --- | --- | --- | --- | --- | --- |
| I^2^ residuals | 0.705 | 0.454 | 0.163 | -0.321 | -0.027 |
| P^3^ residuals | -0.147 | 0.125 | 0.559 | 0.673 | -0.177 |
| M^1^ residuals | -0.254 | 0.314 | -0.389 | 0.147 | 0.705 |
| I_2_ residuals | 0.406 | -0.705 | -0.333 | 0.246 | -0.011 |
| P_3_ residuals | -0.351 | -0.382 | 0.446 | -0.579 | 0.173 |
| M_1_ residuals | -0.358 | 0.194 | -0.447 | -0.165 | -0.664 |


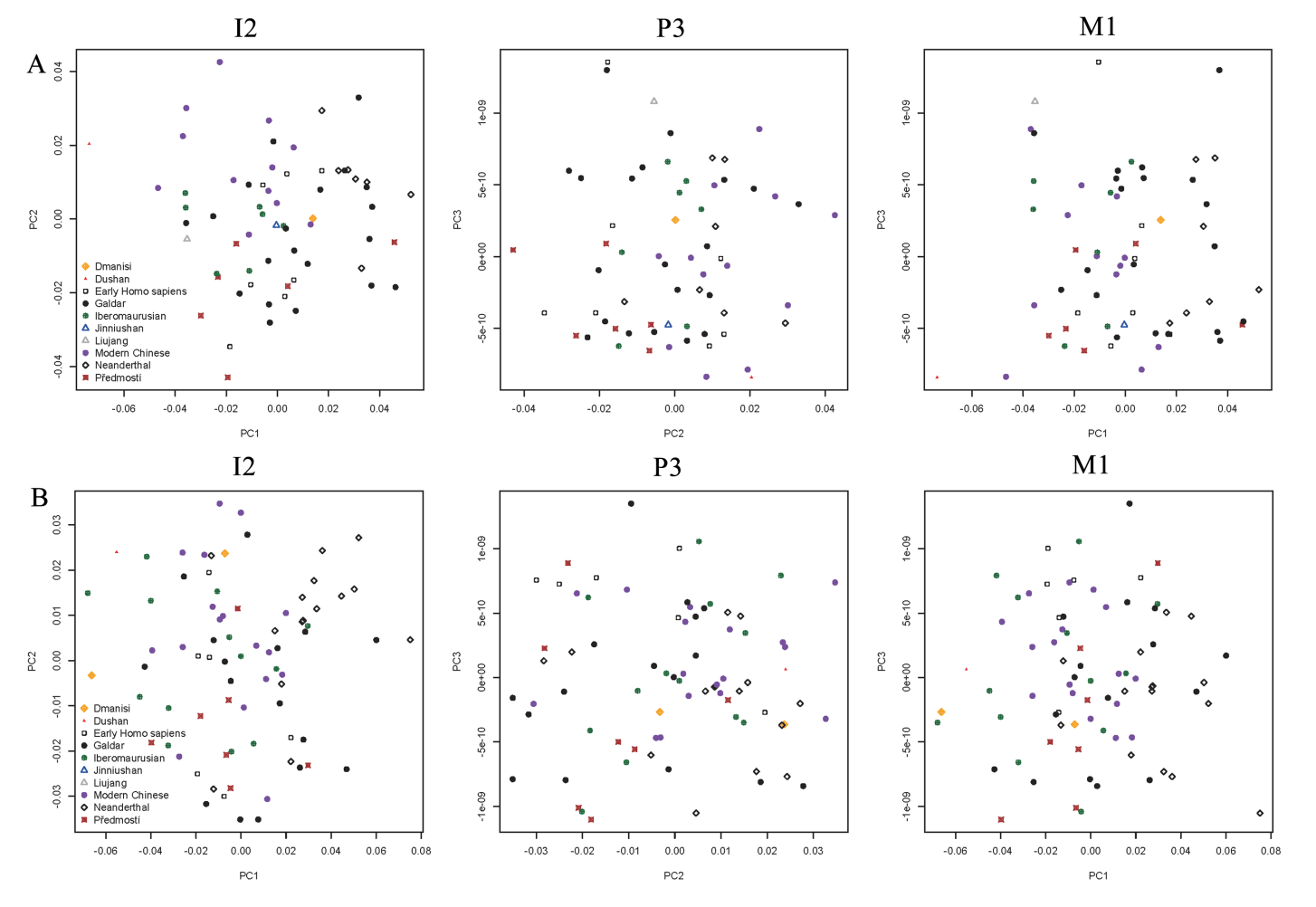


SI-Fig. 6. Principal component analysis of maxillary teeth. PC1 accounts for 69% of total variance (A); principal component analysis of mandibular teeth. PC1 accounts for 72% of total variance (B).


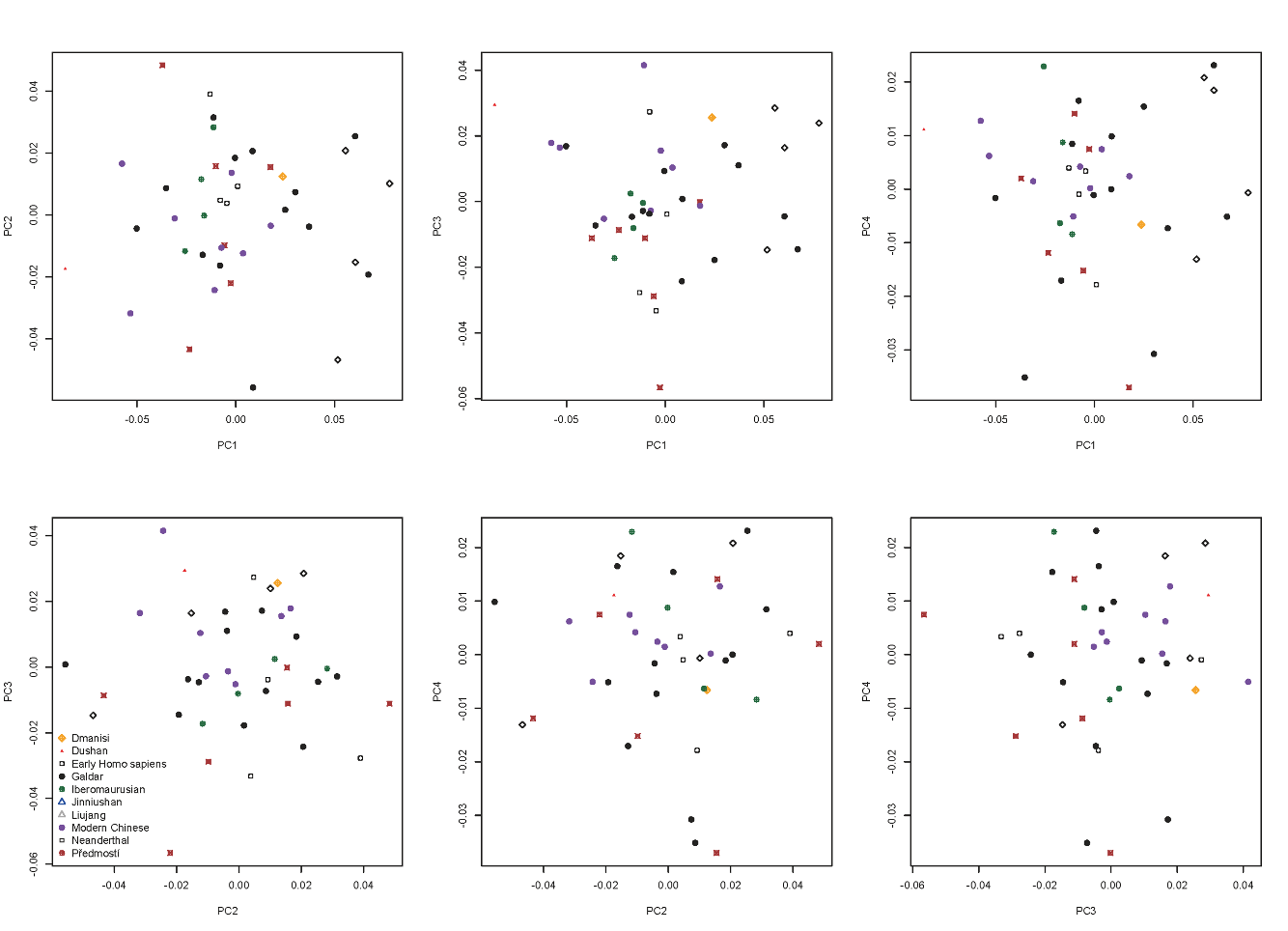


SI-Fig. 7. Principal Component Analysis of specimens with both maxillary and mandibular teeth. Cumulative proportion of variance accounted for by Principal components: PC1: 51%; PC2: 71%; PC3: 86%; PC4: 94%.

**SI-5 Morphological descriptions of individual teeth**

Six small fragments of Dushan 1 mandible are preserved. All of them are affected by some type of chemical/taphonomical erosion so it is difficult to identify some key morphological features. The largest fragment includes the right I_2_-M_1_ in-situ. In this fragment, the mental foramen is placed below P_4_ and opens towards posterior. The thickness of the corpus at the level of the M_1_ is 15.0 mm. This fragment is broken at the level of the central keel of the symphysis, showing a marked incurvatio mandibulae and a deep mental fossa. The wall of the bone projects outwards suggesting a remarkable bony chin. Internally, the wall of the symphysis is almost vertical, and lacks a planum alveolare. No other feature is visible in this fragment. Another small fragment preserves the right M_3_ in-situ. A short (about 6.0 mm) and horizontal retromolar area is observed. A subparallel mylohyoid line is barely marked.

**5.1 Upper left and right central incisors (left I^1^, right I^1^) (**Fig. 1, SI-Figs. 3, 9 and 10**)**

Both the left and the right I^1^s are in excellent condition, except for the roots that are shortened because of some type of chemical/taphonomic reason. In buccal and lingual views the shape of the crown is an asymmetric trapezoid, with the incisal edge ascending distally. In occlusal view, the labial surfaces of both I^1^s show weak (left I^1^) and trace (right I^1^) convexity (ASUDAS grade 1 and 2 respectively.) (27, 33). From the lingual aspect both crowns are shovel-shaped (ASUDAS shovel shape grade 4), with the mesial marginal ridge being more pronounced than the distal one. At the EDJ, the shovel shape defines a small but deep triangular fossa. Although occlusal wear has erased some of the lingual features at the OES, there are several short grooves running to the basal region of the crown. These grooves correspond at the EDJ with up to two pronounced ridges without free apex (tuberculum dentale ASUDAS grade 4). In addition, the central and the marginal ridges are slightly elevated at the labial surface.

The roots are robust, with a rounded section, wider at the buccal than at the lingual side. The roots are abnormally shortened by a process of likely taphonomic process.

**5.2 Upper left and right lateral incisor (LI^2^, RI^2^) (**Fig. 1, SI-Figs. 3, 9 and 10**)**

Like the two I^1^s, both left and right I^2^s of Dushan 1 are in excellent condition except for a small enamel chipping at the left incisal corner of the left I^2^.

In buccal and lingual views, the crown is an asymmetric trapezoid with the mesial side being longer than the distal one. Unlike I^1^s, the labial surfaces only show traces of convexity (grade 1 of ASUDAS). Dental wear has erased most of the lingual morphological features but both I^2^s show an asymmetrical shovel shape (ASUDAS grade 4), where the mesial marginal ridge is more pronounced than the distal one. The marginal ridges converge at the basal region in a marked tuberculum dentale without free apex (ASUDAS grade 4). There are one and three lingual grooves run in the lingual surface of the left and the right I^2^s, respectively, which correspond with two and three finger-like projections at the EDJ. Also at the EDJ, a deep lingual fossa is present in both antimeres. At the labial surface, the marginal ridges are slightly elevated potentially corresponding with a double-shoveling grade 1. Finally, the left I^2^ presents a well-developed interruption groove that runs mesially into the root and can be also identified at the EDJ. Both roots are thin and narrow, with triangular section and a tip that deviates distally.

**5.3 Upper left and right canine (LC**^1^**, RC**^1^**) (**Fig. 1, SI-Figs. 3, 9 and 10**)**

Both the left and the right upper canines of Dushan 1 are generally well preserved although some enamel alterations in the left one obscure the identification of some features.

From the buccal view, the crowns are a slightly asymmetrical pentagon and the surface is smooth. The lingual marginal ridges are well developed (shovel shape ASUDAS grade 4). Both upper canines of Dushan 1 exhibit pronounced labial convexity.

Both canines present strong tuberculum dentale that are more pronounced at the dentine level and, in the case of the right canine, exhibits a free apex (grade 5 of ASUDAS). At the EDJ, the essential crests of the canines are well developed at both the labial and the lingual surfaces. At the labial surface of the EDJ, the marginal ridges are marked and separated from the essential ridge by clear longitudinal depressions. Although at the OES, it is not possible to identify it, at the EDJ remarkable distal accessory ridges are present in both antimeres (grade 3 of ASUDAS), so this individual is likely a male.

The roots of both Dushan 1 upper canines look gracile, triangular in section and with thin and sharp apices.

**5.4 Upper left and right third premolar (LP^3^, RP^3^) (**Fig. 1, SI-Figs. 3, 9 and 10**)**

Both the left and right P^3^s are generally well preserved and present complete crowns and roots, although the lingual root of the left P^3^ is resorbed about two thirds.

In buccal view, the crowns of both Dushan 1 P^3^s are trapezoidal and roughly symmetrical, although the distal marginal ridge diverges distally. The crown buccal surface of both P^3^s are relatively smooth but the central ridge is well separated from the mesial and distal marginal ridges by shallow depressions, which are more pronounced in the mesial sides for both P_3_s. The occlusal contour is an asymmetrical pentagon with an expanded buccodistal corner. The lingual cusps are similar in size to the buccal ones, and both apices are mesially displaced with regard to the external contour of the teeth, but relatively centered with regard to each other. The sagittal groove is continuous and U-shaped. No transverse crest is present. Pronounced mesial and distal accessory ridges can be observed in both P^3^s. A large distal accessory marginal tubercle, particularly pronounced at the EDJ can be found in both premolars. Also at the EDJ, the essential ridge of both the buccal (in the left P^3^) and the lingual cusps (in both P^3^s) are bifurcated but this cannot be seen at the enamel because of dental wear. One of the most remarkable features of the dentine at the buccal surface is the basal bulging and the well-demarcated marginal ridges, particularly at the mesial side. The central ridge is also inflated and separated from the marginal ridges by deep depressions. From a lateral view, there is a moderate angle between the basal bulging and the inclined buccal slope of the buccal cusp.

Both P^3^s have a buccal and a lingual root, with high bifurcation. The roots are thin and they narrow strongly towards the tip.

**5.5** **Upper left and right fourth premolar (LP^4^, RP^4^) (**Fig. 1, SI-Figs. 3, 9 and 10**)**

The state of preservation of both P^4^s is generally good except for some small buccal enamel alteration and an enamel chip at the right mesial side of the right P^4^. The roots are also shortened by taphonomic processes for both P^4^s so their original length and robusticity cannot be assessed. From the buccal surface the crown is an asymmetric trapezoid and the surface is smooth. On the occlusal aspect the contour is oval-shaped with a slightly expanded buccal half. The lingual cusp is smaller than the buccal one and both are separated by a continuous U-shaped sagittal groove. Both cusps’ tips are mesially deviated with regard to the external contour but centered with regard to each other. Dental wear has erased most of the features of interest at the OES, but they can be clearly assessed at the dentine level. At the EDJ, the essential crests of the lingual cusps are slightly bifurcated for both P^4^s but the bifurcated essential crest is more pronouncedly developed at the buccal cusp of left P^4^. A distal accessory ridge is present in both P^4^s. A wide but slight elevation at the distal margin could correspond with a distal marginal accessory tubercle and next to the lingual cusp tip there is an elevation that could correspond with a mesial one. As with P^3^s, the marginal ridges and the central ridges at the buccal surface are marked and separated by deep depressions. From the lateral side, there is a moderate basal bulging that creates a marked angle with the buccal slope of the buccal cusp.

The roots are bifurcated into a buccal and a lingual root close to the cement-enamel junction. The roots look narrow but they are affected by some type of chemical erosion that alters their original aspect.

**5.6 Upper left and right first molar (LM^1^, RM^1^) (**Fig. 1, SI-Figs. 3, 9 and 11**)**

Both left and right M^1^s of Dushan 1 are in good condition, except some enamel chippings at the buccal surface of the right M^1^ and at the occlusal aspect of the protocone. Dental wears make it difficult to identify some of the key occlusal traits at the enamel.

The occlusal outlines of both Dushan 1 M^1^s are approximately squared. Both the M^1^s display the four main cusps and a well-developed cusp 5 (ASUDAS grades 3) delimited by a V-shaped groove. Both teeth exhibit medium-sized metacones (ASUDAS grade 3) and large hypocones (ASUDAS grade 4). The crista obliqua is notched by the central groove. A small enamel extensions is observed on the buccal side of the left M^1^ (grade 1 of ASUDAS).

The occlusal aspect of the EDJ of both M^1^s is remarkably complicated by the expression of several interconnected ridges, tubercles and undulations. Similar to the upper premolars, there is a basal bulging at the buccal surface of both M^1^s that is particularly pronounced in the case of the left M^1^ creating a marked angle with the steep buccal slope of the buccal cusps. On the lingual surface of both M^1^s, there is an irregular groove that extends from the base of the protocone to the hypocone and could correspond with either a high degree of Carabelli’s expression which is not visible at the OES or to a pronounced lingual cingulum.

**5.7 Upper left and right second molar (LM^2^, RM^2^) (**Fig. 1, SI-Figs. 3, 9 and 11**)**

Both M^2^s are well-preserved except for the root resorption present in the right M^2^.

The occlusal outline is a bucco-lingually elongated oval-shaped where the four cusps and a pronounced and well-delimited C5 (ASUDAS grade 4) are present. At the EDJ, the C5 presents on the right side a distinct cusp tip. The metacone and the hypocone are relatively small (ASUDAS grade 3). At the OES, the crista obliqua is interrupted by the central groove whereas at the EDJ it is continuous and low.

The morphology at the EDJ level is less complicated than that of M^1^ but there are still numerous ridges, tubercles and undulations. At the EDJ both molars present a deep cleft in the lingual aspect of both cusps and bulges outwards being remarkably pronounced in the left side. This structure presents the shape of a pronounced cleft that traverses the lingual aspect of the protocone and extends distally onto the hypocone. This structure could correspond to a Carabelli’s grade 7 or more likely, to a cingulum-like structure, as it does not show free apex or a cingulum-protocone crest (see Ortiz et al., 2012). At the buccal aspect of the EDJ we find again the relative bulging of the basal part of the crown in contrast with the steep and more depressed buccal aspect of the cusps. All these features are not visible at the OES.

Both molars have three roots, two buccal and a lingual radical that separate close to the cement-enamel junction. There is a small enamel extension at the point of bifurcation between both buccal radicals. The lingual radical looks longer and robust, but the buccal radicals seem to be taphonomically thinned and shortened.

**5.8 Upper right third right molar (RM^3^) (**Fig. 1, SI-Figs. 3, 9 and 11**)**

The crown is very well preserved and the roots are shortened by a taphonomic process. The four main cusps and a well-developed cusp 5 are present. The metacone and the hypocone are small (ASUDAS grade 2) and the hypocone occupies a peripheral lingual position. The C5 is large (ASUDAS grade 4) and well delimited. The occlusal surface at the EDJ is relatively simple and five clear cusp tips, including that of the C5, can be identified. At the lingual aspect of the protocone at the EDJ, there is a deep groove that extends from the distal arm of the hypocone dentine horn onto the lingual surface of the protocone. Similar to the M^1^ and the M^2^, this feature could correspond to a Carabelli’s trait grade 4 or, due to its more shelf-like aspect, to a lingual cingulum. None of these features are identified at the OES.

This tooth presents three root radicals, one lingual and two buccal. The buccal ones are shortened by taphonomic processes.

**5.9 Lower left and right central incisor (LI_1_, RI_1_) (**Fig. 1, SI-Figs. 3, 12 and 13**)**

Both lower central incisors are in an excellent stage of preservation. From the buccal aspect the contour is triangular with the distal margin slightly more divergent than the mesial. From the occlusal aspect, the surface is almost flat (labial convexity ASUDAS grade 1) and no shovel shape no tuberculum dentale is present. At the EDJ, the morphology is also simple and the slight elevation of the marginal ridges at the labial surface, particularly for the left side, correspond with a grade 1 double shoveling. The root is single and gracile, with a cylindrical section that narrows towards the tip.

**5.10 Lower left and right lateral incisors (**Fig. 1, Fig. 1, SI-Figs. 3, 12 and 13**)**

The state of preservation is excellent except for the apical third of the left I_2_ root missing. The general aspect is simple and gracile. From the labial aspect, the crown is a symmetric triangle and in the lingual aspect the surface is smooth and featureless. From the occlusal aspect, the dentine reveals a slightly thickened marginal ridge (shovel shape ASUDAS grade 1) and at the EDJ, there are traces of double shovel shape (ASUDAS grade 1). In the left I_2_, the central ridge is also slightly elevated. The root is single and gracile, with an oval section that narrows towards the tip.

**5.11 Lower left and right canine (LC_1_, RC_1_) (**Fig. 1, Fig. 1, SI-Figs. 3, 12 and 13**)**

The state of preservation is excellent except for a minimal loss at the root apex of the left canine. From the labial aspect, the crown is an asymmetrical trapezoid where the distal margin is longer and more divergent than the mesial. The external aspect is smooth except for clear depressions (grooves) that separate the mesial and distal marginal ridges from the essential ridge that can be observed on the labial surface in both C_1_s.

From the occlusal aspect no tuberculum dentale can be seen (ASUDAS grade 0). At the OES a faint shovel shape (ASUDAS grade 1) is barely perceptible by palpation, although the marginal ridges are more pronounced at the EDJ. Also at the EDJ, the marginal ridges (particularly the mesial) and the central ridge are elevated. On the left canine, a small distal accessory ridge can be identified at the EDJ but no at the OES. At the labial surface of the EDJ, the expression of two vertical grooves that separate the elevated central ridge from the mesial and distal marginal ridges are more pronounced than at the OES.

Externally, both canine roots are single and present a wide and shallow developmental groove separating their buccal and lingual radicals throughout the whole root length. The left C_1_ shows a bifid tip. The Micro-CT reconstruction reveals that the root canal of both C_1_s bifurcates into two independent canals at the middle third that coalesce again in the apical third (Fig. 1, and SI-Figs. 12, 13 and 17).

**5.12 Lower left and right third premolar (LP_3_, RP_3_) (**Fig. 1, SI-Figs. 3, 12, 13 and 17**)**

The state of preservation is excellent except for the apical third of the left P_3_ root that is missing.

The occlusal contour is an asymmetrical oval, due to the expression of a distolingual talonid. The buccal cusp is larger than the lingual cusp and both are separated by a continuous and short sagittal groove. The tips of both main cusps are centered with regard to each other. At the EDJ, an accessory lingual cusp can be identified with its apex in the distolingual aspect of the talonid. Apart from the essential ridge a long mesial accessory ridge and a short distal accessory ridge can be identified.

From the buccal aspect, the outline is a slightly asymmetric trapezoid, where the mesial margin diverges more than the distal. The essential ridge is inflated and separated from the pronounced mesial marginal ridge by a clear triangular depression. At the EDJ, there is a strong basal bulging in the buccal surface. The occlusal half of the buccal surface is strongly inclined towards lingual, defining a strong angle between the basal cingulum and the steep buccal surface of the buccal cusp. The marginal ridges, particularly the mesial one, are remarkably elevated and they separate from the slightly pronounced essential ridge by deep depressions (Fig. 3). The accessory distal ridge is also demarcated in the buccal surface.

Both P_3_s of Dushan 1 are three rooted, with distobuccal (DB), mesiobuccal (MB) and distolingual (DL) canals (DB+MB+DL). In the right P_3_ the buccal and lingual radicals are joined by a thin lamina of cementum at the distal side and only fully separate at the apex whereas in the mesial side they are fully separated throughout the whole length. At the disto-buccal surface of the buccal radical, a clear and deep groove further divides the buccal radical into distobuccal (DB) and mesiobuccal (MB) radicals. For the left P_3_, two buccal roots fully separate from the second third. Because the two thirds of the DL radical are missing in the left P_3_ we cannot assess if they separate. The reconstructed root canals show that both P_3_s of Dushan 1 have three separate canals (Fig. 6). From the CT section we can see that the right P_3_ displays a buccal plate that separates from the lingual radical in the first third of the root and that the buccal plate separates into independent bucco-mesial and bucco-distal canal in the lower third (Fig. 6). In the left side, three independent canals can be seen from the second third of the root. The root and canal form of both Dushan P_3_s can be classified as the type 3-C-1 as defined by Moore et al (55).

**5.13 Lower left and right fourth premolar (LP_4_, RP_4_) (**Fig. 1, SI-Figs. 3, 12, 13 and 17**)**

Both P_4_s are very well preserved with only some surface alteration of the enamel in the left one.

From the occlusal aspect, the contour is approximately rectangular. Both main cusps are separated by a continuous U-shaped groove. The tip of the lingual cusp is mesial with regard to the buccal one. Distal to the lingual cusp, and separated by a short groove, there is an additional lingual cusp or almost the same size as the metaconid. From the buccal aspect, the contour is trapezoidal in shape. The buccal enamel of the left P_4_ is damaged, but on the right side the surface is relatively smooth, although shallow mesial and distal depressions delimitate the central and the marginal ridges.

At the EDJ, the essential ridge of the left buccal cusp is prominent and single, whereas in the right one it bifurcates from the tip in two branches. Distal to the buccal essential ridge there is a distal accessory ridge, well pronounced in the case of the left P_4_ and barely perceivable in the right antimere. In both premolars, the additional lingual cusps are very well developed. The tips of the metaconid and the accessory distal cusp are strongly expressed and particularly the metaconid is a very tall cusp.

Like in the P_3_s, a strong basal bulging can be identified at the buccal surface of the EDJ and from it, strongly inclined and elevated mesial and marginal ridges develop. The marginal ridges and the essential ridge are strongly marked and well separated by deep triangular buccal depressions (Fig. 5).

In both antimeres, the root is single and short, and with an oval section. In the case of the left P_4_, it ends in a bifid tip. But a pronounced groove can be observed at about two-thirds of the mesial root surface of the right P_4_ (grade 3 of ASUDAS).

**5.14 Lower left and right first molar (LM_1_, RM_1_) (**Fig. 1, SI-Figs. 3, 12 and 13**)**

Both molars are generally well preserved except for the general taphonomic alteration that has shortened and thinned the roots of the left M_1_. Both antimeres present the five main cusps with a particularly large hypoconid that protrudes in the external outline of the tooth. The cusps are arranged in a Y-pattern. The hypoconulid (cusp 5) is large (ASUDAS grade 4). Occlusal wear prevents the identification of a possible C6 and C7 at the OES. There are no signs of middle or distal trigonid crest. The buccal surface is smooth at the OES and an enamel extension can be identified.

In contrast with the apparent simplicity of the enamel surface, the occlusal surface of the EDJ is remarkably complicated with the expression of numerous crenulations, ridges and undulations of the surface. Distal to the hypoconulid cusp there is an elevation of the distal marginal ridge that could correspond with the expression of a C6. However, this feature cannot be identified at the OES because of the wear degree. Also at the EDJ, there is a deep groove extending from the mesial to the distal aspect of the buccal side of the protoconid that would correspond with an ASUDAS grade 5 of protostylid. It is noteworthy that the expression of this protostylid groove is pronounced, extending not only across the protoconid but also across most of the upper buccal portion of the hypoconind in both M_1_s. The buccal surface is strongly inclined so it is projected onto the occlusal view.

The right molar is three rooted. The mesial root comprises a buccal and a lingual radical that do not bifurcate, whereas the disto-buccal and the disto-lingual roots bifurcate close to the cement-enamel junction. These roots are thin and narrow strongly towards the tip. The roots of the left molar are damaged but in the CT section the same four canals can be observed in the preserved part.

**5.15 Lower left and right second molar** **(LM_2_, RM_2_) (**Fig. 1, SI-Figs. 3, 12 and 13**)**

The teeth are in good state of preservation with only damage at the apical third of the roots of both antimeres.

The cusps are present and arranged in a “+” groove pattern in the right M_2_ and a “X” groove pattern in the left M_2_. There is a large C5 (ASUDAS grade 4) in the left antimere whereas in the right the identification is obscured because of the dental wear. At the OES, there are no signs of anterior fovea, C6, C7, middle or distal trigonid crest. On the buccal surface of the protoconid of the right side there is a short groove extending from the buccal groove that could correspond with a protostylid grade 5. However, the presence of a round caries at this level prevents its identification on the left antimere. An enamel extension is present in both teeth.

At the EDJ, there are clear C5 and C6 in the left antimere, but no traces in the right one. The morphology of the occlusal surface is less complicated than in the M_1_, but there are still many crenulations, ridges and undulation. Discontinuous middle and distal trigonid crests can be seen. In both teeth, the basal part of the buccal surface is strongly bulged. Its upper limit is marked by a linear depression that extends onto the protoconid and connects a deep V-shaped groove without free apex. This, together with a small ridge at the mesiobuccal aspect of the hypoconulid conform a cingulum-like protostylid of approximate grade 5 or 6.

The molars display buccal and lingual plate-like roots that are highly bifurcated. The buccal root has two canals without external bifurcation whereas the lingual root is single throughout the whole length.

**5.16 Lower left and right third molar (LM_3_, RM_3_) (**Fig. 1, SI-Figs. 3, 12 and 13**)**

Both the teeth are very well preserved except for the chemical erosion that affects the roots and has shortened their length. Five main cusps are present and arranged in an “X” pattern. The C5 is large (ASUDAS grade 4) and buccally oriented, and no signs of C6 or C7 are present. There are no middle trigonid crest and distal trigonid in the left M_3_. However, the right antimere presents a short groove in the metaconid, paralleling to the central groove that could correspond with a grade 1 distal trigonid crest. On the buccal surface of the protoconid of both M_3_s, a marked secondary groove extends mesially from the buccal groove and the groove separating the hypoconid from the hypoconulid is also pronounced. As a whole, these grooves conform a protostylid grade 5. At the OES, an enamel extension can be also seen.

At the EDJ, there is a clear basal bulging at the buccal surface so this side projects strongly onto the occlusal view. The protostylid is very pronounced at the EDJ in the shape of a deep V groove at the buccal aspect of the protoconid, a light horizontal depression making the upper limit of the basal bulge, and a short distal groove at the distal aspect of the hypoconid. These features are more pronounced in the right antimere (Fig. 5). Apart from this, the EDJ of these teeth is relatively simple.

Both molars have a mesial and a distal plate-like root that bifurcate below the enamel junction. The mesial one bifurcates into a mesiobuccal and a mesiolingual canal from the second third, but there is no external division. The distal root has only one canal.

**5.17 Non-metric PCA (LM_3_, RM_3_) (**SI-Fig. 8**)**

In the present study, PCA was conducted based on eight non-metric traits for Dushan 1 and comparative samples of East Asian fossil hominins and recent modern humans. These eight non-metric traits are usually regarded showing evolutionary or taxonomic values (28-29, 33, 39, 48), and exhibit more pronounced expression in Dushan 1. As shown in SI-Table 9, first and second PCs explain the 46.4% and 32.7% of the total variations respectively. In SI-Fig. 8, Dushan 1 falls at the right side of the PC1 axis together with archaic hominins and early modern humans. Both Dushan 1 and fossil hominins are clearly separated from the recent modern humans that stay at the left side of PC1 axis. But in PC2, Dushan 1 exhibits the resemblance to the recent modern humans.

SI-Table 9. Component loadings and variance proportions of the PCA analysis based on eight non-metric traits of Dushan 1 and comparative samples.

| Variables | Component loadings | |
| --- | --- | --- |
|  | PC-1 | PC-2 |
| I^1^ labial convexity | .474 | -.019 |
| P^3^ bifurcated essential crest | -.067 | .042 |
| P^3^ mesial and distal accessory ridge | -.016 | .031 |
| P^3^ accessory marginal tubercle | .379 | .071 |
| M^1^ Carabelli’s cusp | .402 | .221 |
| M_1_ protostylid | .131 | .488 |
| P^3^ crown buccal vertical groove | -.225 | .501 |
| Complicated occlusal morphology at EDJ for molars | .442 | -.125 |
| Proportion of the variance (%) | 46.4 | 32.7 |
| Cumulative proportion (%) | 46.4 | 79.1 |


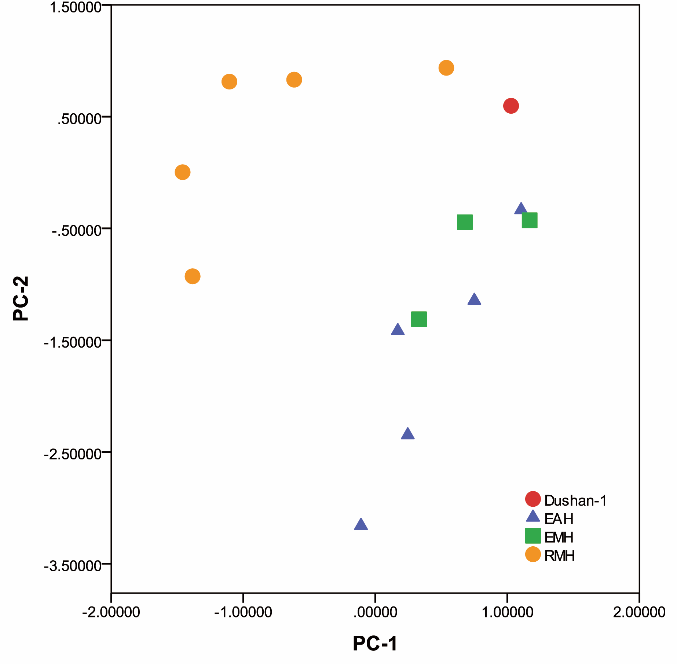


SI-Fig. 8. Results of the PCA based on eight non-metric traits for Dushan 1 and comparative samples. EAH=East Asian archaic *Homo*; EMH=Early modern humans; RMH= Recent modern humans.

**SI-6 Figures and Tables**


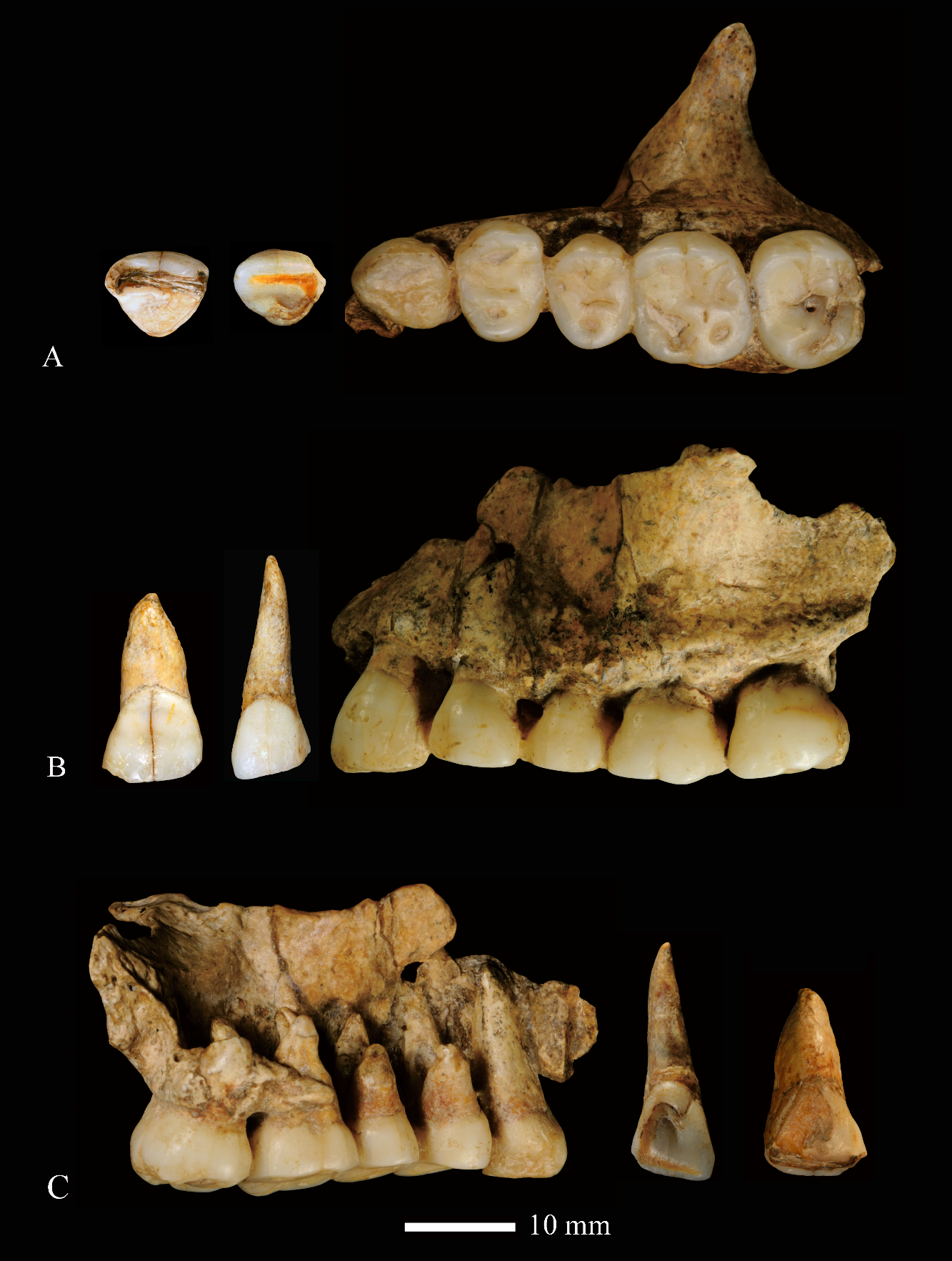


SI-Fig 9. Left upper teeth (I^1^ to M^2^) of Dushan 1. A: occlusal view; B: buccal view; C: lingual view.


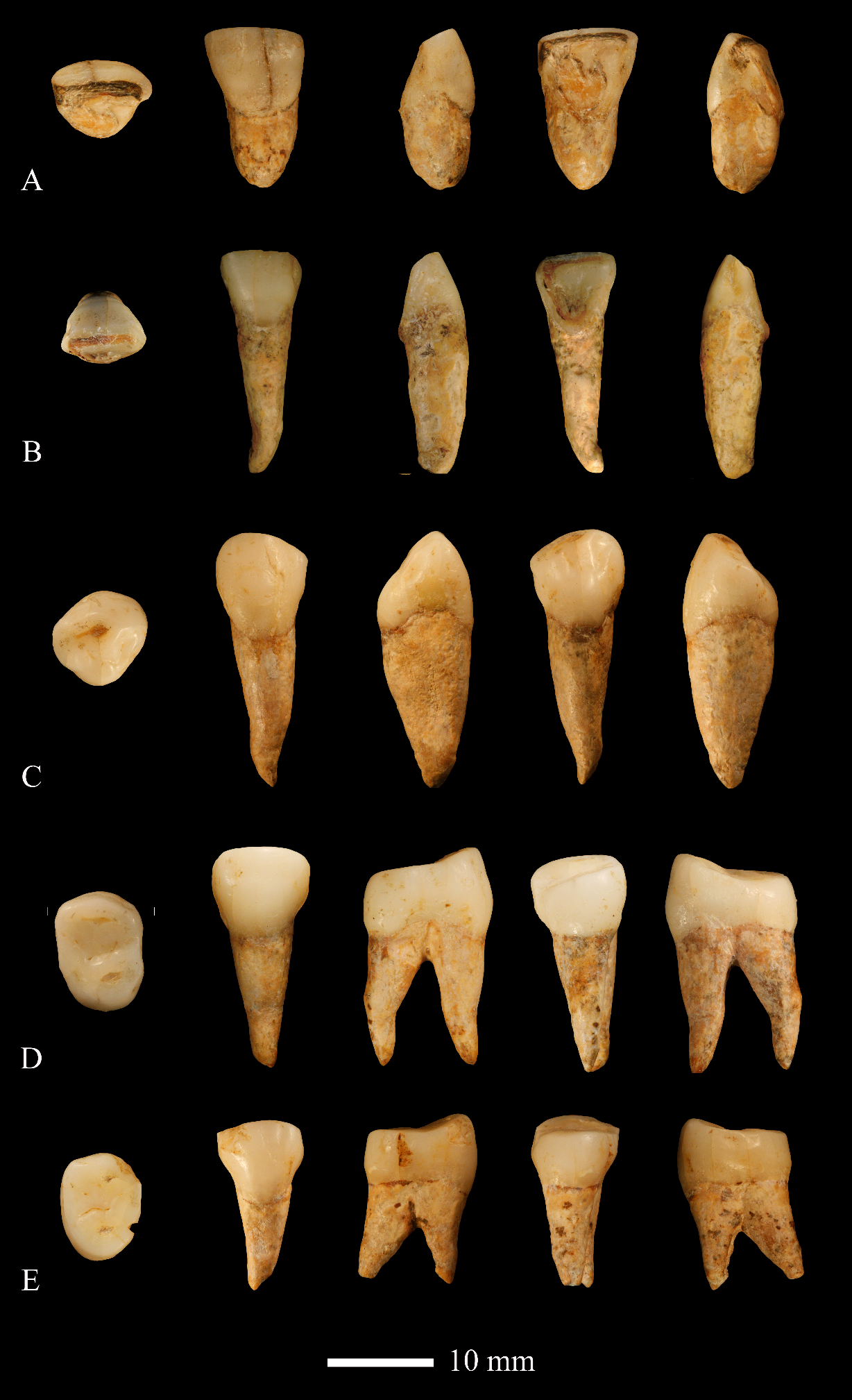


SI-Fig. 10. Right upper teeth (A-E: I^1^, I^2^, C^1^, P^3^, P^4^) of Dushan 1. From left to right: occlusal, buccal, mesial, lingual and distal views.


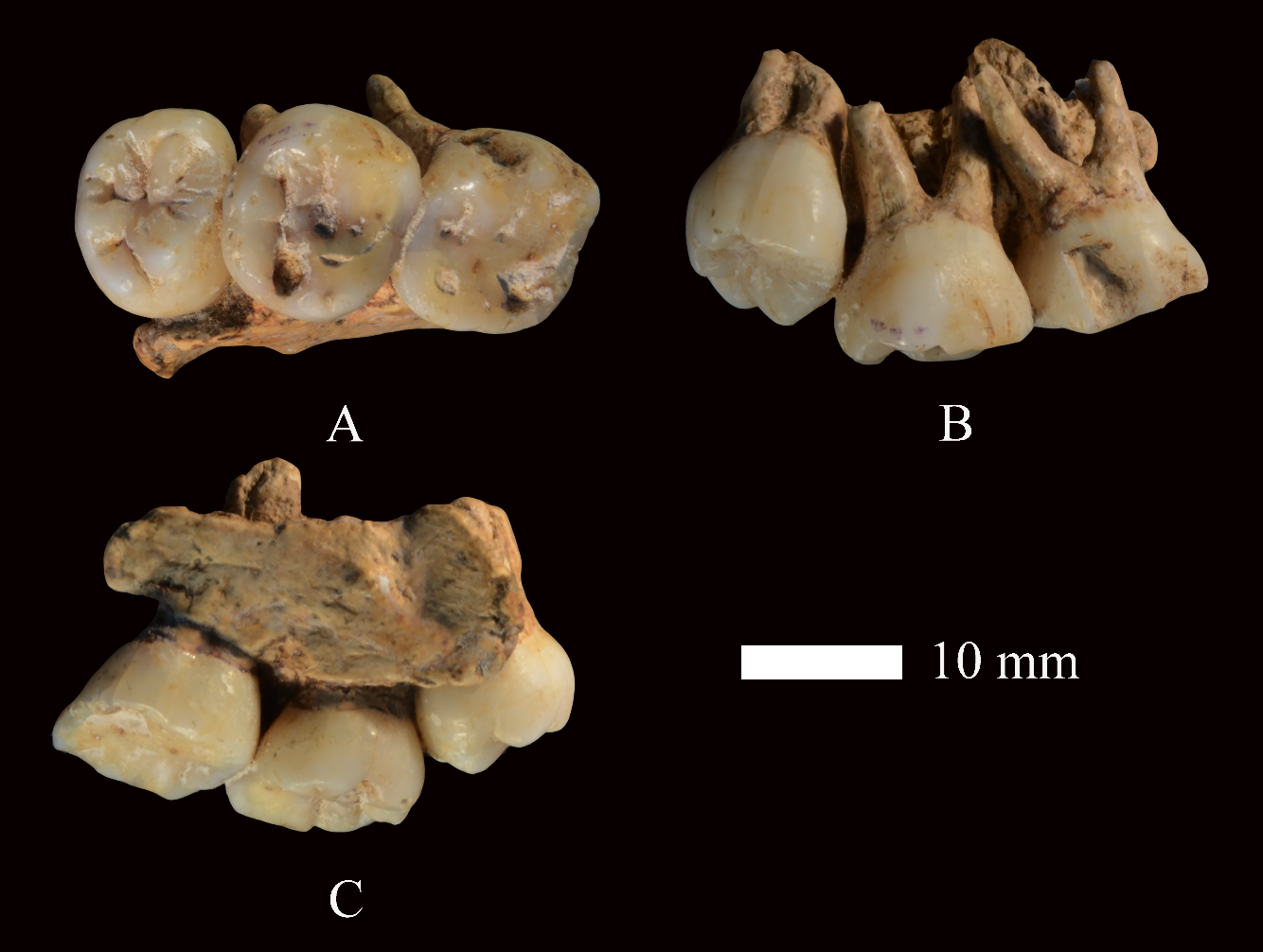


SI-Fig 11. Right upper teeth (M^1^, M^2^, M^3^) of Dushan 1. A: occlusal view; B: buccal view; C: lingual view.


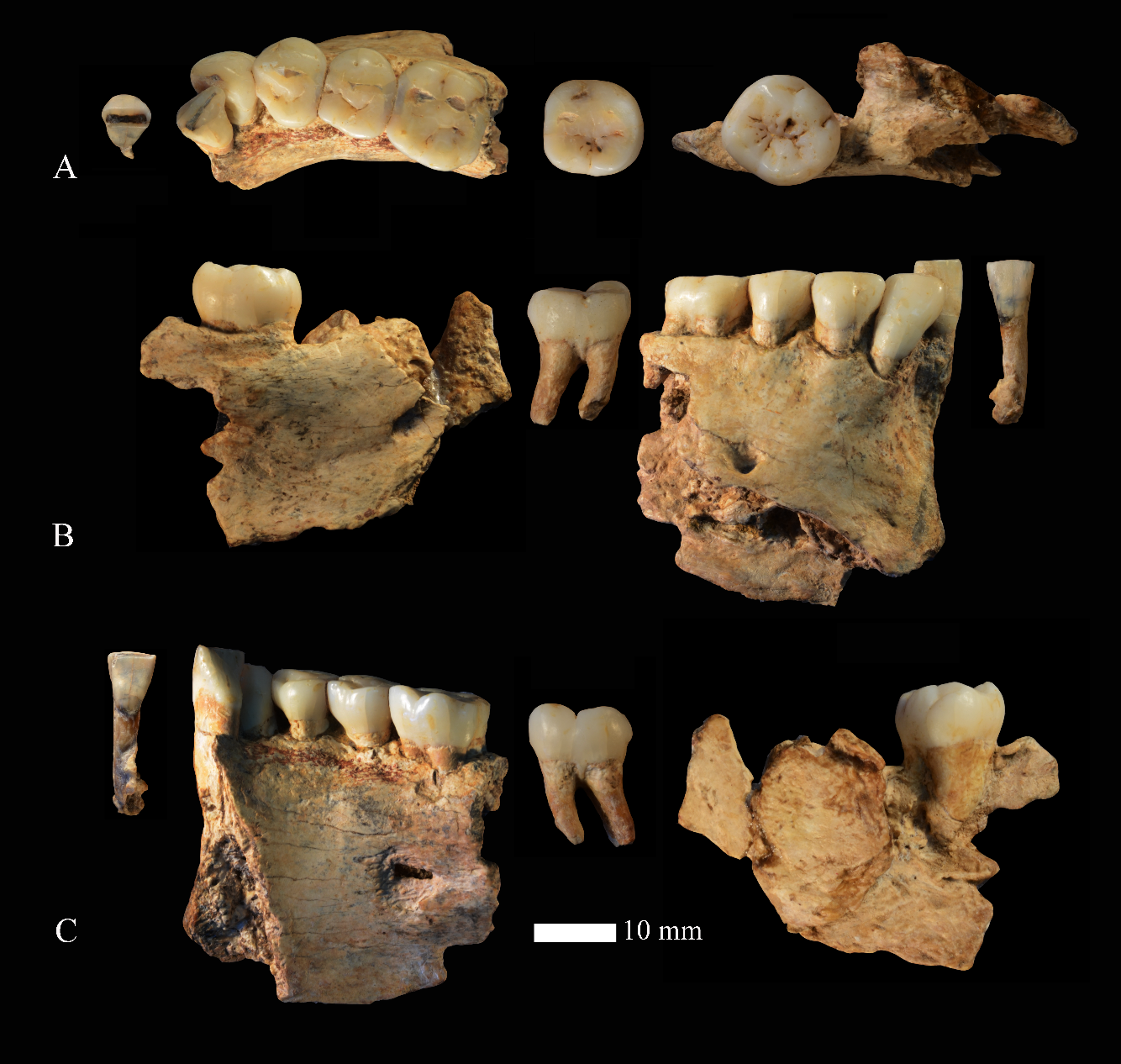


SI-Fig 12. Right lower teeth (I_1_ to M_3_) of Dushan 1. A: occlusal view (from left to right: I_1_ to M_3_); B: buccal views (from right to left: I_1_ to M_3_); C: lingual view (from left to right: I_1_ to M_3_).


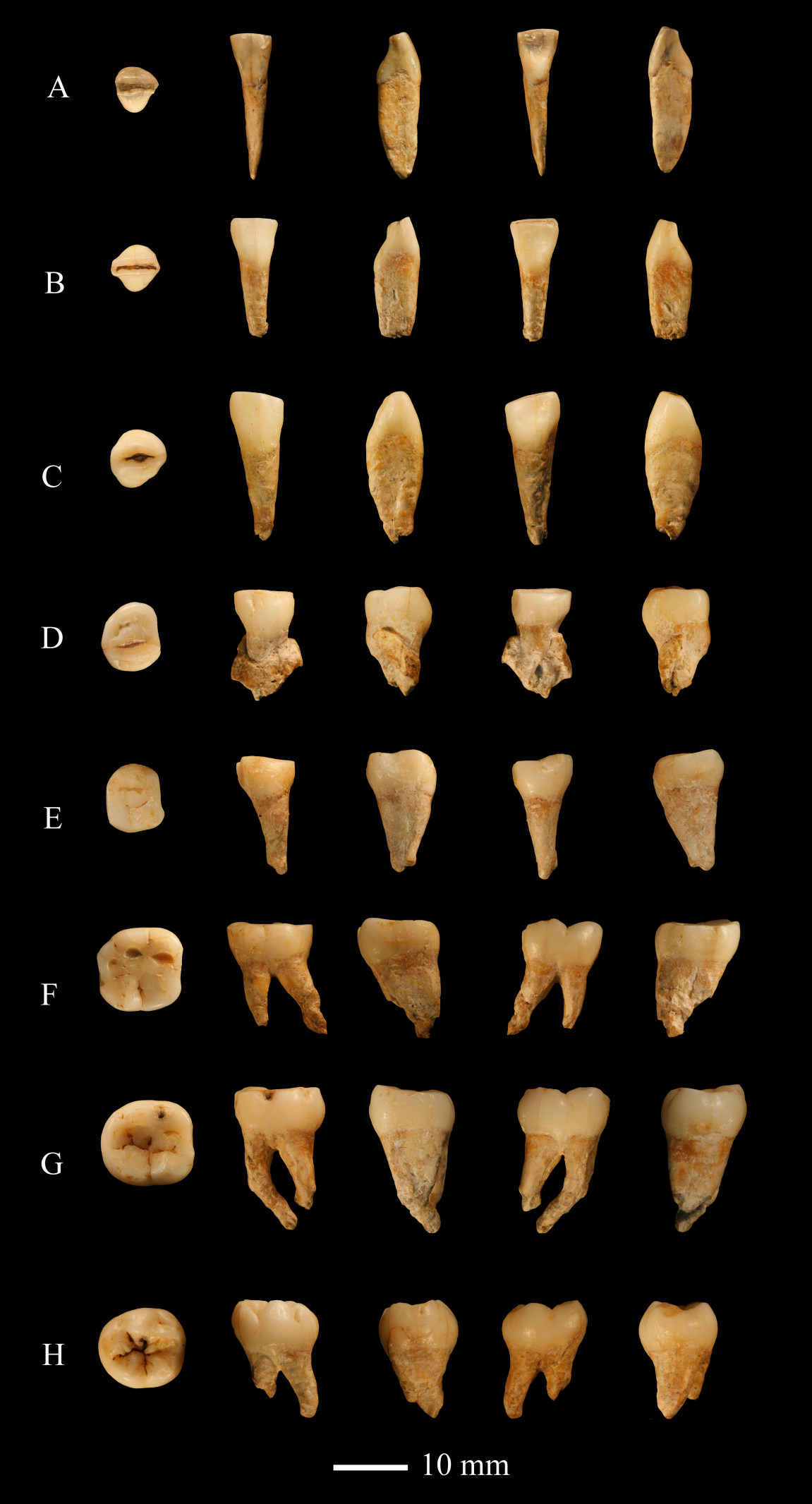


SI-Fig. 13. Left lower teeth of Dushan 1 (rows A-H: I_1_ to M_3_; columns from left to right: occlusal, buccal, mesial, lingual and distal views).

**
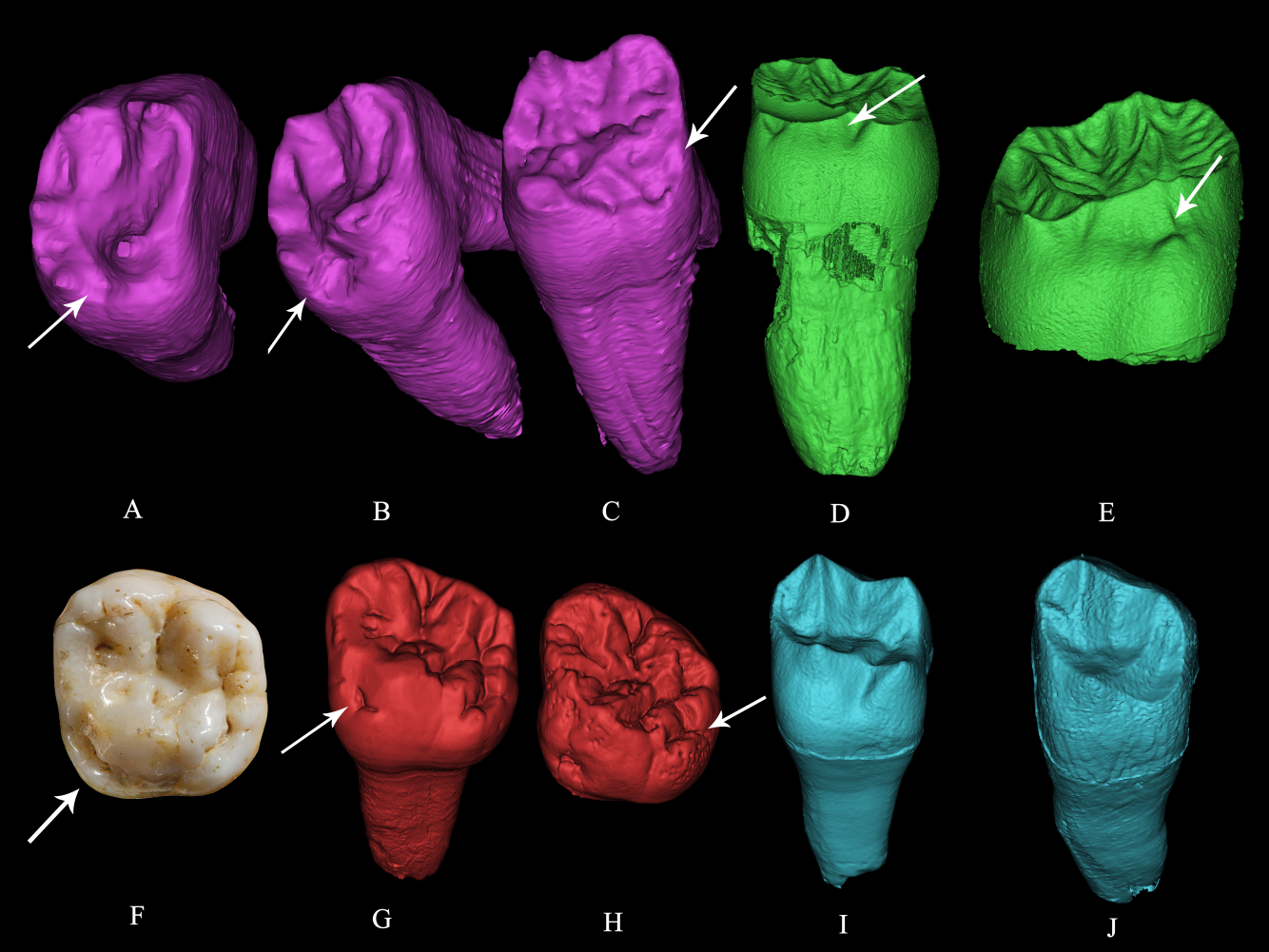
**

SI-Fig. 14. Carabelli's trait-like complex identified in the upper molars of Dushan 1 and comparative samples. A: Dushan 1 right M3; B: Dushan 1 right M2; C: Dushan 1 right M1; D: Hexian left M1(PA836); E: Hexian right M2(PA837); F: Xujiayao left M1(PA1480-5, OES); G: Xujiayao left M1(PA1480-5); H: Xujiayao left M2(PA1480-6); I: Modern human M1; J: Modern human M2.

(Buccal side facing upper side. Except image F, all the images are created from Micro-CT scanning. All the images are not scaled).


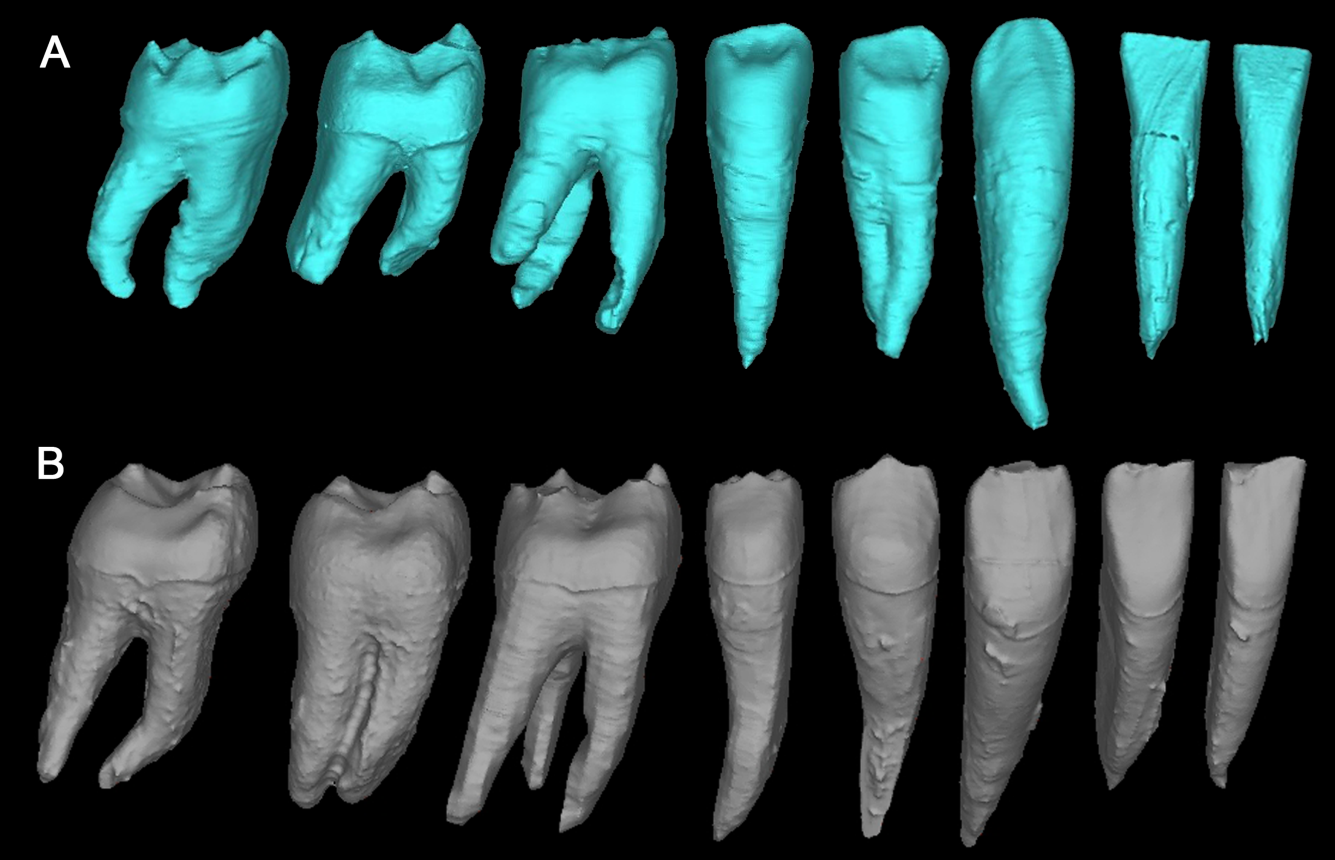


SI-Fig. 15. Buccal EDJ views of right lower dentitions for Dushan 1 (A) and modern human (B). (All the EDJ images are not scaled).


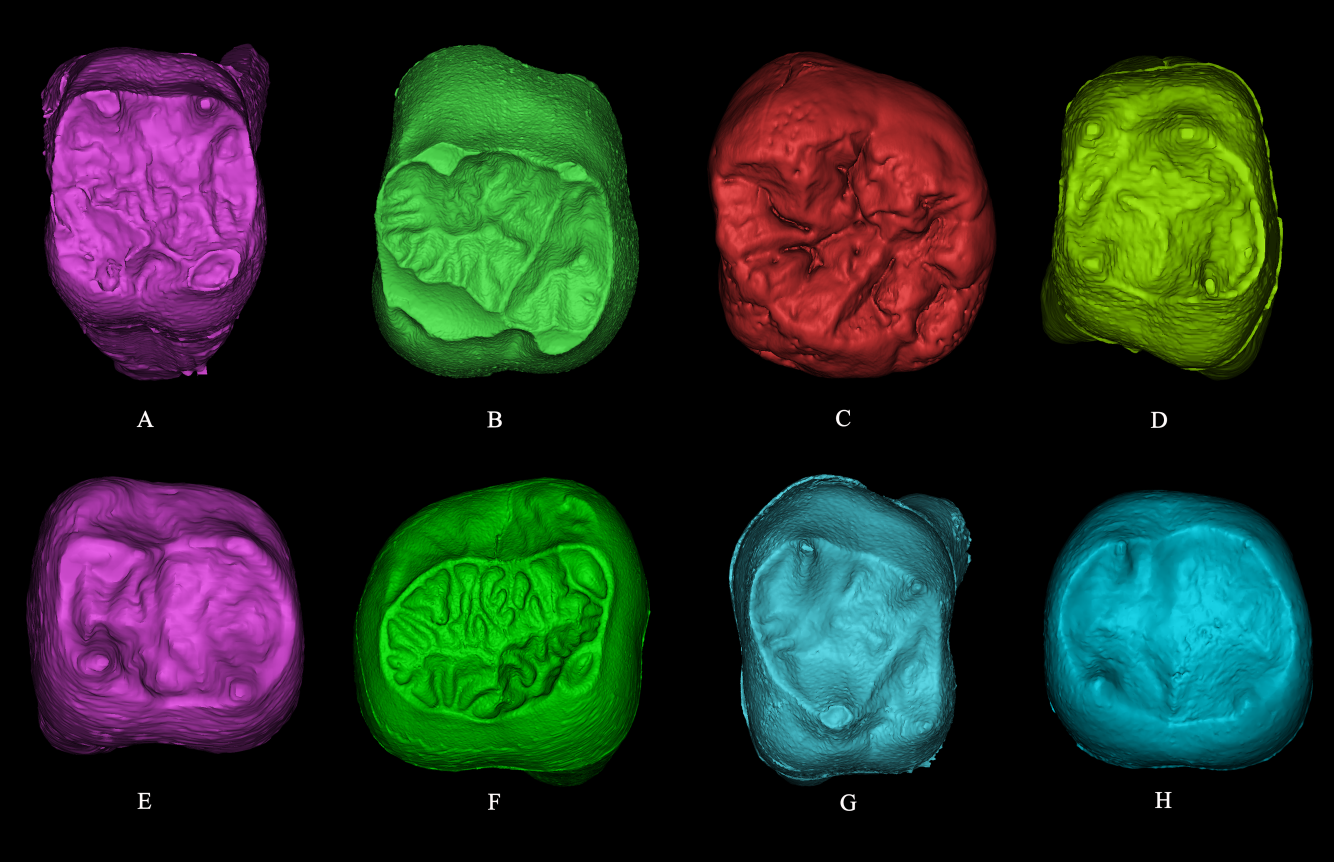


SI-Fig. 16. Comparison of molar occlusal morphologies at EDJ between Dushan 1 and comparative samples. A: Dushan 1 right M^1^; B: Hexian(PA836) left M^1^; C: Xujiayao (PA1480) left M_1_; D: Daoxian(DX36) left M^2^; E: Dushan 1 right M_2_; F: Zhoukoudian(PA70) left M_2_; G: modern human left M^1^; H: modern human right M_2_.

**
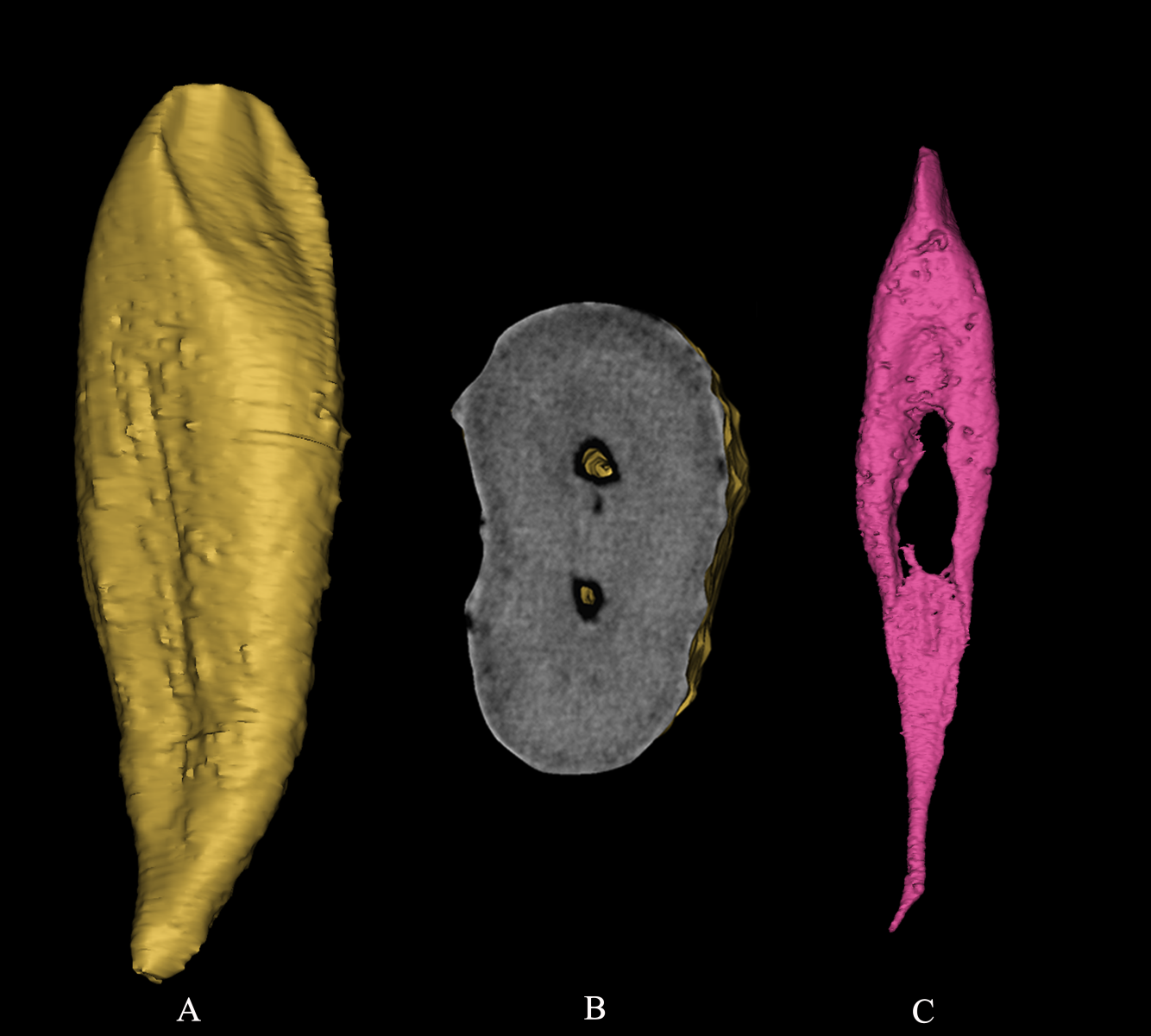
**

SI-Fig. 17. Left lower canine of Dushan 1. A: External view; B: Cross section of the root at middle portion based Micro-CT; C: Root canal reconstructed from Micro-CT.

SI-Table 10. Tooth size of Dushan 1 and comparative samples

|  | I1 | | I2 | | C | | P3 | | P4 | | M1 | | M2 | | M3 | |
| --- | --- | --- | --- | --- | --- | --- | --- | --- | --- | --- | --- | --- | --- | --- | --- | --- |
|  | MD | BL | MD | BL | MD | BL | MD | BL | MD | BL | MD | BL | MD | BL | MD | BL |
| **Maxillary teeth** | | | | | | | | | | | | | | | | |
| **Dushan 1** | **9.3** | **7.1** | **8.1** | **6.8** | **9.0** | **9.1** | **8.4** | **11.4** | **8.0** | **10.8** | **12.6** | **12.1** | **12.1** | **13.3** | **10.7** | **13.2** |
| Maludong |  |  |  |  |  |  |  |  |  |  |  |  |  |  |  | 12.5 |
| Longlin |  |  |  |  |  |  |  |  |  | 11.0 |  | 11.7 |  |  |  |  |
| EAAH | 10.4  8.3-11.7  0.8  N=15 | 8.2  6.4-9.4  0.7  N=14 | 7.8  7.0-8.3  0.5  N=6 | 7.7  6.0-8.2  0.9  N=6 | 9.4  8.5-10.8  0.7  N=11 | 9.9  8.8-10.6  0.5  N=11 | 8.6  7.4-9.3  0.6  N=18 | 11.8  10.0-13.0  1.0  N=18 | 8.2  7.2-9.2  0.6  N=17 | 11.5  10.3-12.7  0.8  N=17 | 11.7  10.2-13.8  0.9  N=22 | 13.0  11.0-14.2  0.9  N=22 | 11.2  10.2-12.5  0.7  N=12 | 13.4  12.2-15.5  1.0  N=12 | 9.5  8.2-11.4  0.8  N=12 | 11.5  9.2-13.7  1.3  N=12 |
| EAEMH | 8.4  8.1-8.7  0.2  N=7 | 7.2  6.5-7.8  0.6  N=7 | 7.3  6.4-8.2  0.6  N=6 | 7.2  6.7-7.9  0.5  N=6 | 8.1  7.4-9.0  0.5  N=13 | 8.8  6.9-10.4  0.9  N=13 | 7.4  6.2-8.0  0.6  N=10 | 10.2  9.8-10.7  0.3  N=10 | 6.8  6.2-7.5  0.6  N=8 | 9.7  8.5-10.6  0.7  N=8 | 10.6  9.5-12.7  0.8  N=31 | 11.9  10.2-13.8  0.9  N=31 | 10.1  9.0-11.3  0.7  N=13 | 12.0  9.9-14.2  1.2  N=12 | 9.4  8.1-11.5  1.1  N=16 | 11.0  9.7-12.0  1.6  N=16 |
| GLEMH | 9.3  8.1-11.5  0.9  N=30 | 7.7  6.5-8.8  0.7  N=30 | 7.5  6.4-9.7  0.8  N=25 | 7.2  6.0-8.3  0.6  N=25 | 8.2  6.7-10.0  0.7  N=37 | 9.0  6.9-10.8  0.9  N=37 | 7.4  6.2-8.9  0.6  N=37 | 10.0  8.5-11.1  0.6  N=37 | 7.0  6.0-7.9  0.6  N=38 | 9.9  8.5-11.3  0.7  N=39 | 10.9  9.5-12.7  0.8  N=81 | 12.2  10.2-14.0  0.8  N=81 | 10.5  8.2-12.6  0.9  N=45 | 12.3  9.9-14.2  1.0  N=44 | 9.5  8.1-11.5  0.8  N=37 | 11.5  9.7-13.5  0.9  N=37 |
| NEA | 9.5  6.8-10.9  1.0  N=28 | 8.6  7.5-9.9  0.6  N=28 | 8.1  7.1-9.3  0.6  N=30 | 8.5  6.0-9.9  0.7  N=30 | 8.6  6.9-10.0  0.7  N=37 | 9.9  8.8-11.4  0.6  N=37 | 7.8  6.3-9.3  0.7  N=32 | 10.6  9.2-11.8  0.7  N=32 | 7.4  6.3-8.8  0.7  N=33 | 10.4  9.0-11.7  0.6  N=33 | 11.5  9.9-13.5  0.9  N=37 | 12.1  10.1-14.2  0.8  N=37 | 10.8  9.0-13.1  1.0  N=36 | 12.6  11.4-14.2  0.7  N=36 | 9.8  8.4-11.4  0.8  N=25 | 12.3  10.4-13.8  0.8  N=25 |
| EARMH | 8.7  8.0-9.9  0.4  N=37 | 7.1  6.4-7.8  0.3  N=37 | 7.2  6.5-8.2  0.4  N=40 | 6.5  5.7-7.4  0.4  N=40 | 7.8  6.6-8.7  0.5  N=46 | 8.3  7.2-9.9  0.5  N=46 | 7.2  6.4-8.5  0.4  N=66 | 9.3  6.7-10.6  0.6  N=66 | 7.0  6.0-8.1  0.4  N=75 | 9.4  8.3-10.5  0.5  N=75 | 10.6  9.2-12.4  0.6  N=62 | 11.6  10.1-13.3  0.6  N=62 | 9.8  8.7-11.2  0.5  N=78 | 11.6  10.3-13.4  0.7  N=78 | 9.2  7.8-10.2  0.6  N=37 | 11.1  9.1-12.5  0.8  N=37 |
| GLRMH | 8.9  7.8-10.8  0.6  N=119 | 7.3  6.2-8.5  0.4  N=145 | 7.0  4.9-8.9  0.6  N=146 | 6.6  5.5-7.7  0.5  N=169 | 7.9  6.3-9.5  0.5  N=250 | 8.5  6.3-10.4  0.6  N=268 | 7.4  6.0-9.1  0.5  N=344 | 9.6  6.7-12.0  0.7  N=343 | 7.1  5.9-8.5  0.5  N=334 | 9.5  6.9-11.4  0.7  N=347 | 10.8  8.6-12.4  0.7  N=450 | 11.9  9.9-14.2  0.7  N=448 | 10.0  7.7-13.1  0.8  N=451 | 12.0  9.5-14.6  0.9  N=452 | 9.2  5.0-11.6  0.9  N=315 | 11.4  6.5-14.6  1.1  N=315 |
| **Mandibular teeth** | | | | | | | | | | | | | | | | |
| **Dushan 1** | **5.9** | **6.3** | **6.7** | **6.8** | **7.9** | **8.4** | **8.3** | **10.0** | **8.4** | **10.3** | **11.7** | **11.8** | **12.2** | **11.5** | **12.2** | **11.3** |
| Maludong |  |  |  |  |  |  |  |  |  |  |  |  |  | 11.9 |  | 11.6 |
| Longlin |  |  |  | 6.7 |  | 8.4/7.6 |  | 9.3/9.5 |  |  |  |  |  |  |  | 10.7/10.3 |
| EAAH | 6.4  6.0-6.7  0.3  N=5 | 6.4  5.8-6.8  0.4  N=5 | 7.0  6.3-7.7  0.5  N=8 | 7.1  6.4-8.4  0.6  N=8 | 8.4  7.9-9.0  0.5  N=9 | 8.9  8.2-10.4  0.9  N=9 | 8.5  7.9-9.8  0.6  N=14 | 9.6  8.2-10.7  0.7  N=14 | 8.6  7.2-9.9  0.7  N=10 | 10.1  8.0-11.1  0.9  N=10 | 12.9  9.9-14.6  1.2  N=15 | 12.0  10.1-13.6  0.9  N=15 | 13.0  11.1-15.3  1.4  N=22 | 12.6  10.1-16.1  1.4  N=22 | 12.1  10.0-14.0  1.5  N=10 | 11.5  10.0-13.6  1.2  N=10 |
| EAEMH | 4.7  4.3-5.5  0.7  N=3 | 5.8  5.7-6.1  0.2  N=3 | 5.2  4.3-6.1  0.9  N=5 | 6.4  6.0-7.1  0.4  N=5 | 7.1  6.3-7.8  0.5  N=9 | 8.4  6.6-9.2  0.9  N=9 | 7.1  5.7-8.8  1.0  N=7 | 8.3  7.5-8.9  0.4  N=7 | 7.0  5.8-8.7  0.9  N=7 | 8.8  8.2-10.0  0.9  N=7 | 11.1  10.2-12.1  0.6  N=23 | 10.8  9.7-11.6  0.5  N=23 | 11.4  9.9-14.0  0.5  N=22 | 10.7  9.3-12.0  0.9  N=23 | 10.9  9.7-13.0  0.7  N=16 | 10.5  9.4-12.6  0.7  N=17 |
| GLEMH | 5.7  4.3-6.7  0.8  N=23 | 6.4  5.7-7.3  0.5  N=23 | 6.3  4.3-7.9  0.9  N=30 | 6.9  5.9-9.0  0.7  N=30 | 7.3  6.1-8.8  0.7  N=33 | 8.6  6.6-10.0  0.7  N=33 | 7.2  5.7-8.8  0.7  N=36 | 8.6  7.0-9.6  0.6  N=36 | 7.4  5.8-8.7  0.7  N=28 | 8.7  8.0-10.0  0.5  N=28 | 11.5  10.0-13.1  0.8  N=68 | 11.0  9.2-12.7  0.6  N=68 | 11.3  9.2-14.0  0.9  N=64 | 10.8  8.7-12.7  0.8  N=65 | 11.2  8.5-13.2  1.1  N=44 | 10.6  8.0-12.7  0.9  N=45 |
| NEA | 5.8  4.6-8.0  0.8  N=16 | 7.4  6.8-8.2  0.4  N=16 | 6.6  5.3-7.5  0.5  N=24 | 7.9  7.3-8.8  0.4  N=24 | 7.9  6.7-8.8  0.5  N=36 | 9.1  7.5-10.3  0.7  N=36 | 7.8  6.6-9.1  0.6  N=38 | 9.1  8.0-10.3  0.6  N=38 | 7.5  5.9-9.4  0.7  N=29 | 9.2  7.7-10.5  0.6  N=29 | 11.6  10.1-13.6  0.8  N=53 | 11.0  10.0-12.9  0.6  N=53 | 12.0  10.5-14.0  0.8  N=42 | 11.2  9.8-12.4  0.6  N=42 | 11.7  9.7-12.7  0.7  N=32 | 11.0  8.4-13.4  1.0  N=32 |
| EARMH | 5.3  4.7-6.0  0.3  N=28 | 5.6  5.0-6.3  0.3  N=28 | 6.3  5.9-6.9  0.3  N=20 | 6.2  5.5-6.8  0.4  N=20 | 7.1  6.5-8.1  0.3  N=39 | 7.7  6.6-9.0  0.4  N=39 | 7.2  6.4-8.0  0.4  N=65 | 7.9  6.7-9.0  0.5  N=65 | 7.2  6.5-8.6  0.5  N=42 | 8.2  7.5-10.1  0.5  N=42 | 11.4  10.0-12.7  0.5  N=42 | 10.7  9.8-11.5  0.5  N=42 | 11.1  10.0-12.5  0.6  N=51 | 10.4  9.3-11.6  0.5  N=51 | 11.0  8.2-13.1  1.0  N=77 | 10.3  7.2-12.2  0.9  N=77 |
| GLRMH | 5.5  4.7-6.4  0.4  N=82 | 5.8  4.9-7.4  0.4  N=105 | 6.2  5.2-7.5  0.4  N=105 | 6.3  5.0-7.6  0.5  N=133 | 7.1  5.4-8.4  0.5  N=197 | 7.8  6.3-9.8  0.6  N=213 | 7.4  5.7-9.2  0.5  N=273 | 8.1  6.5-10.2  0.6  N=276 | 7.4  6.2-9.1  0.5  N=257 | 8.5  6.4-10.1  0.6  N=260 | 11.8  9.7-14.0  0.7  N=314 | 10.9  8.9-13.0  0.7  N=317 | 11.3  9.5-13.6  0.8  N=330 | 10.6  8.6-13.4  0.7  N=331 | 11.2  8.2-13.8  1.0  N=302 | 10.4  7.2-12.9  0.8  N=302 |

EAAH= East Asian Middle Pleistocene archaic *Homo*; EAEMH= East Asian Early Modern Human; GLEMH= Global Early modern humans; NEA= Neanderthals; EARMH= East Asian recent modern humans; GLRMH=Global recent modern humans

SI-Table 11. Comparisons of the non-metric and linear metric traits of Dushan 1 and comparative samples

|  |  | Dushan 1 | East Asia  archaic *Homo* | East Asia  EMH | East Asia  RMH | Java  *H. erectus* | Neanderthal | Global  *H. sapiens* |
| --- | --- | --- | --- | --- | --- | --- | --- | --- |
| Trait comparisons (Frequency and specimen number) | | | | | | | | |
| 1 | I^1^ Shoveling | 2/2 | 100%(15/15) | 71.4% (5/7) | 97.6%(81/83) |  | 90.5%  N=21 | 3%-90%  Average:60.1%  N=1356 |
| 2 | I^1^ Double shoveling | 0/2 | 0%(0/14) | 14.3%(1/7) | 40.5%(79/195) |  |  | 0%-70%  Average:35.4%  N=1221 |
| 3 | I^1^ Labial convexity | 1/2 | 92.8%(13/14) | 42.9%(3/7) | 9.6%(8/83) |  | 100%  N=21 | 30.8%  N=91 |
| 4 | I^1^ Tuberculum dentale | 2/2 | 93.3%(14/15) | 71.4%(5/7) | 25.6%(20/78) |  | 90.5%  N=21 | 26.6%  N=90 |
| 5 | C^1^ Tuberculum dentale | 2/2 | 100%(15/15) | 53.8%(7/13) | 38.0%(76/200) |  | 100%  N=21 | 20.4%  N=112 |
| 6 | I^2^ Interruption groove  present | 1/2 | 0%(0/7) | 0%(0/6) | 43.6%(92/211) |  |  | 10.4%-65.0%  N=5693 |
| 7 | C^1^Distal accessory ridge | 2/2 | 100%(11/11) | 25%(2/8) | 57.7%(75/130) |  | 43.7%  N=16 | 55.0%  N=856 |
| 8 | C_1_ Distal accessory ridge | 1/2 | 100%(9/9) | 0%(0/4) | 36.8%(14/38) |  | 78.8%  N=18 | 6%  N=133 |
| 9 | P^3^ Transverse crest present | 0/2 | 17.7%(3/17) | 20%(1/5) | 4.2%(3/71) | 33.3%(3/9) | 6.7% N=15 | 2%(6/283) |
| 10 | P^4^ Transverse crest present | 0/2 | 21.4%(3/14) | 25%(1/4) | 2.9%(2/70) | 50%(4/8) | 12.4% N=16 | 2%(5/279) |
| 11 | P_3_ Transverse crest present | 0/2 | 100%(10/10) | 50%(2/4) | 78.6%(63/80) | 100%(4/4) |  | 82%(176/215) |
| 12 | P_4_ Transverse crest present | 0/2 | 12.5%(1/8) | 33.3%(1/3) | 42.5%(31/73) | 67%(4/6) |  | 18%(40/220) |
| 13 | P^3^ Bifurcated essential crest | 2/2 | 100% (15/15) | 0%(0/4) | 44.2%(23/52) |  | 53.3% N=15 | 11.1% N=126 |
| 14 | P^4^ Bifurcated essential crest | 2/2 | 100% (16/16) |  | 55.3%(26/47) |  | 78.6% N=14 | 9.4% N=106 |
| 15 | P_3_ lingual cusp number  (≥ 2 cusps) | 2/2 | 100%(10/10) | 75%(3/4) | 62.3%(48/77) |  | 81.5%  N=27 | 35.4%  N=150 |
| 16 | P_4_ lingual cusp number  (≥ 2 cusps) | 2/2 | 100%(9/9) | 100%(3/3) | 79.3%(218/275) |  | 100%  N=27 | 71.7%  N=2272 |
| 17 | P^3^ Mesial or distal  accessory ridge | 2/2 | 93.3% (14/15) | 50%(2/4) | 43.3%(26/60) |  | 36.4% N=11 | 23.4%-57.0%  N=749 |
| 18 | P^4^ Mesial or distal  accessory ridge | 2/2 | 93.8% (15/16) |  | 67.6%(46/68) |  | 41.7% N=12 | 33.0%-80.2%  N=749 |
| 19 | P_3_ Mesial or distal  accessory ridge | 2/2 | 100%(10/10) | 25%(1/4) | 52.0%(40/77) |  | 90% N=20 | 11.9% N=13521 |
| 20 | P_4_ Mesial or distal  accessory ridge | 2/2 | 100%(8/8) | 33.3%(1/3) | 46.6%(34/73) |  | 70% N=20 | 23% N=115 |
| 21 | P^3^ Accessory marginal  tubercle | 2/2 | 93.3% (14/15) | 25%(1/4) | 19.2%(10/52) |  |  |  |
| 22 | P^4^ Accessory marginal  tubercle | 2/2 | 93.8% (15/16) |  | 14.9%(7/47) |  |  |  |
| 23 | P^3^ Two-rooted premolars | 2/2 | 88.9% (8/9) | 0%(0/3) | 47.8%(22/46) |  |  | 4.9-66.7%  N=11443 |
| 24 | P^4^ Two-rooted premolars | 2/2 | 40% (2/5) | 0%(0/6) | 4.3%(2/47) |  |  | ? |
| 25 | P_3_ Two-rooted premolars | 2/2 | 0%(0/9) | 0%(0/5) | 21.7%(10/46) | 50%(3/6) |  | 4%(26/599) |
| 26 | P_4_ Two-rooted premolars | 2/2 | 0.0%(0/7) | 0%(0/4) | 0.0%(0/73) | 57%(4/7) |  | 3%(15/570) |
| 27 | Three-rooted P_3_ | 2/2 | 0%(0/9) | 0%(0/5) | 0%(0/46) |  |  | 0%(0/599) |
| 28 | P_3_ Tomes’s Root | 2/2 |  |  | 64.8% (142/219) |  |  | 19.9% N=1371 |
| 29 | Five-cusped M^1^ | 2/2 | 20%(6/20) | 35.3%(6/17) | 36.8%(25/68) |  |  | 26.1% N=2070 |
| 30 | Five-cusped M^2^ | 2/2 | 33%(3/9) | 14.3%(1/7) | 17.9%(22/79) |  |  | 38.5% N=208 |
| 31 | Five-cusped M^3^ | 1/1 | 54.5%(6/11) | 33.3%(3/9) | 18.8%(12/64) |  |  | 26.8% N=157 |
| 32 | M^1^ Metacone (≥grade 2) | 2/2 | 100%(20/20) | 100%(17/17) | 100%(89/89) |  | 100%, N=23 | 99.6% N=216 |
| 33 | M^2^ Metacone (≥grade 2) | 2/2 | 100%(9/9) | 100%(7/7) | 100%(94/94) |  | 100%, N=12 | 100% N=224 |
| 34 | M^3^ Metacone (≥grade 2) | 1/1 | 100%(11/11) | 100%(9/9) | 95.5%(63/66) |  | 94.4%, N=18 | 96.9% N=159 |
| 35 | M^1^ Hypocone (≥grade 2) | 2/2 | 100%(20/20) | 100%(17/17) | 100%(90/90) |  | 95.7%, N=23 | 99.2% N=217 |
| 36 | M^2^ Hypocone (≥grade 2) | 2/2 | 100%(9/9) | 100%(7/7) | 95.6%(87/91) |  | 100%, N=12 | 88.6% N=2712 |
| 37 | M^3^ Hypocone (≥grade 2) | 1/1 | 100%(11/11) | 88.9%(8/9) | 65.2%(43/66) |  | 64.7%, N=17 | 77.4% N=159 |
| 38 | M^1^ Enamel extensions | 1/2 | ?%(?/20) | 21.4%(3/14) | 72.5%(50/69) |  |  | 0%-54.6%  N=11711 |
| 39 | M^1^ Carabelli’s trait | 2/2 | 45%(9/20) | 21.4%(3/14) | 9.7%(27/279) | 100%, N=2 | 50%  N=20 | 1.9-36.0%  N=2426 |
| 40 | Four cusped M_1_ | 0/2 | 0%(0/13) | 0%(0/11) | 1.2%(1/81) | 0%(0/9) | 0%, N=34 | 3%(9/268) |
| 41 | Four cusped M_2_ | 1/2 | 0%(0/19) | 20%(2/10) | 21.7%(13/60) | 0%(0/15) | 0%, N=24 | 58%(163/279) |
| 42 | M_1_ Lower molar cusp 6 | 2/2 | 30.7%(4/13) | 18.2%(2/11) | 34.4%(21/61) |  | 48.3%, N=29 | 33.5% N=1974 |
| 43 | M_2_ Lower molar cusp 6 | 1/2 | 57.9%(11/19) | 0%(0/10) | 36.7%(22/60) |  | 54.2%, N=24 | 2.8% N=151 |
| 44 | M_3_ Lower molar cusp 6 | 0/2 | 50%(4/8) | 0%(0/7) | 52.5%(42/80) |  | 66.7%, N=15 | 22.3% N=121 |
| 45 | M_1_ Lower molar cusp 7 | 0/2 | 23.1%(3/13) | 10%(1/11) | 2.3%(2/87) |  | 69%, N=29 | 8.2% N=2584 |
| 46 | M_2_ Lower molar cusp 7 | 0/2 | 42.1%(8/19) | 0%(0/10) | 1.4%(1/74) |  | 24%, N=25 | 10.3% N=155 |
| 47 | M_3_ Lower molar cusp 7 | 0/2 | 62.5%(5/8) | 0%(0/7) | 4.9%(4/82) |  | 46.5%, N=15 | 21% N=124 |
| 48 | M_1_ protostylid | 2/2 | 53.9%(7/13) | 18.2%(2/11) | 18.3%(63/345) |  | 3.2%  N=31 | 18.7%  N=2362 |
| 49 | M_2_ Lower protostylid | 2/2 | 63.2%(12/19) | 0%(0/9) | 6.8%(5/74) |  | 22.7 N=22 | 11.7% N=154 |
| 50 | M_3_ Lower protostylid | 2/2 | 75%(6/8) | 0%(0/7) | 16.1%(13/81) |  | 38.5% N=13 | 11.8% N=119 |
| 51 | M_1_ Deflecting wrinkle | 2/2 | 61.5%(8/13) | 57.1%(4/7) | 25.0%(21/84) |  | 17.2% N=29 | 4.9%-39.5%  N=4789 |
| 52 | M_2_ Deflecting wrinkle | 2/2 | 63.2%(12/19) | 25%(1/4) | 2.8%(2/71) |  | 26%, N=23 | 0% N=144 |
| 53 | M_3_ Deflecting wrinkle | 0/2 | 50%(4/8) | 42.9%(3/7) | 3.8%(3/80) |  | 6.7%, N=15 | 0% N=117 |
| 54 | M_1_ Middle trigonid crest | 0/2 | 7.7%(1/13) | 0%(0/7) | 19.7%(15/76) | 44% N=9 | 90% N=30 | 13.7% N=102 |
| 55 | M_2_ Middle trigonid crest | 1/2 | 10.5%(2/19) | 0%(0/4) | 23.2%(16/69) | 8% N=13 | 100%, N=23 | 17.6% N=153 |
| 56 | M_3_ Middle trigonid crest | 0/2 | 0%(0/8) | 0%(0/7) | 12.0%(9/75) |  | 94.1%, N=17 | 10.1% N=119 |
| 57 | M_1_ Distal trigonid crest | 0/2 | 15.4%(2/13) | 14.3%(1/7) | 61.5%(48/78) |  | 42.9%, N=28 | 0%-18.7%  N=7047 |
| 58 | M_2_ Distal trigonid crest | 1/2 | 0%(0/19) | 0%(0/4) | 17.1%(12/70) |  | 59.1%, N=22 | 12.7% N=102 |
| 59 | M_3_ Distal trigonid crest | 1/2 | 12.5%(1/8) | 42.9%(3/7) | 16.5%(13/79) |  | 37.5&, N=16 | 11.3% N=151 |
| 60 | Three-rooted M_1_ | 2/2 | 7.7%(1/13) | 0%(0/15) | 24.2%(15/62) |  |  | 0-31.1% |
| 61 | Two-rooted M_3_ | 2/2 | 50%(4/8) | 83.3%(10/12) | 83.7%(41/49) |  |  |  |
| 62 | Complex occlusal morphology at EDJ of molars | 5/5 | 100% (16/16) | 15.2% (5/33) | 5.2% (8/153) |  |  |  |
| 63 | Buccal basal swelling  C^1^  P^3^  P^4^  M^1^  M^2^  M^3^  C_1_  P_3_  P_4_  M_1_  M_2_  M_3_ | 2/2  2/2  2/2  2/2  2/2  2/2  2/2  2/2  2/2  2/2  2/2  2/2 | 54.6%(6/11)  55.6%(10/18)  64.3%(9/14)  63.2%(12/19)  63.6%(7/11)  58.3%(7/12)  72.7%(8/11))  86.7%(13/15)  55.6%(5/9)  88.9%(16/18)  59.1%(13/22)  76.9%(10/13) | 15.4%(2/13)  22.2%(2/9)  22.2%(2/9)  9.7%(3/31)  15.4%(2/13)  14.3%(2/14)  11.1%(1/9)  14.3%(1/7)  16.7%(1/6)  15.8%(3/19)  20%(3/15)  0%(0/14) | 5.8%(1/17)  0%(0/52)  0%(0/47)  25.7%(26/101)  4.9%(2/41)  7.2%(4/55)  0%(0/2)  12.9%(11/85)  2.9%(3/102)  0%(0/129)  0%(0/126)  4.9%(5/103) | 33.3%(1/3) |  | 0.0%(0/207) |
| 64 | Buccal vertical groove  I^1^  I^2^  C^1^  P^3^  P^4^  M^1^  M^2^  M^3^  I_1_  I_2_  C_1_  P_3_  P_4_  M_1_  M_2_  M_3_ | Absent(2/2)  Weak (2/2)  Mod. (2/2)  Strong (2/2)  Strong (2/2)  Strong (2/2)  Strong (2/2)  Strong (2/2)  Weak (2/2)  Weak (2/2)  Mod. (2/2)  Strong (2/2)  Strong (2/2)  Strong (2/2)  Strong (2/2)  Strong (2/2) | 0%  0%  53.8%(7/13)  50%(6/12)  45.5%(5/11)  0%(0/16)  0%(0/13)  0%(0/11)  0%  0%  71.4%(5/7)  72.7%(8/11)  70%(7/10)  0(0/13)  0%(0/19)  0(0/8) | 0%(0/6)  0%(0/6)  8.3%(1/12)  0%(0/10)  0%(0/8)  0%(0/29)  0%(0/11)  0%(0/15)  0%(0/3)  0%(0/5)  0%(0/7)  0%(0/7)  0%(0/7)  0%(0/18)  0%(0/18)  0%(0/18) | 0%(3/31)  0%(3/41)  12.2%(9/74)  26.1%(18/69)  7.6%(5/66)  0%(0/85)  1.1%(1/93)  1.5%(1/66)  0%(0/19)  0%(1/18)  1.4%(1/72)  1.5%(1/68)  6.5%(4/62)  0%(0/129)  6.8%(4/59)  8.5%(6/71) | 67%(6/9)  12%(1/8)  66.6%(2/3)  80%(4/5) |  | 57%(139/245)  31%(67/217)  34%(57/170)  31%(51/164) |
| 65 | Crown buccal vertical groove complex | Present | Absent | Absent | Absent  N=5 |  | Weakly expressed  (La Quina H18) |  |
| 66 | Tooth size proportions  I^1^-C^1^ / P^3^-M^3^ | 0.32 | 0.40 | 0.37 | 0.37 |  | 0.42 | 0.36 |
| 67 | Tooth size proportions  I_1_-C_1_ / P_3_-M_3_ | 0.26 | 0.27 | 0.25 | 0.26 |  | 0.31 | 0.26 |
| 68 | Molar size % increase  M_1_- M_2_ | -0.5 /+2.6  N=2 | Mean=-1.4  - 11.5/ +6.3  N=6 | Mean=1.2  -3.1 / +9.9  N=8 | Mean=-5.0  -14.2 / +4.8  N=174 | +4 / +9  N=5 |  | \| −15 / +5  N=250 \| \| --- \| |
| 69 | Molar size % increase  M_2_- M_3_ | -1.3 / -0.9  N=2 | Mean=-2.8  - 13.1/ +6.3  N=5 | Mean=-3.7  -6.4 / +4.7  N=5 | Mean=-2.6  -18.9 / +18.0  N=124 | -10 / 0  N=5 |  | \| −15 / +14  N=211 \| \| --- \| |

**SI-7-References**

1. Xue, X-X. Human fossil tooth from Luonan, Shaann, and its geological age. Acta Anthrpologica Sinica 6, 284-288 (1987).
2. Chang, C-H., Kaifu, Y., Takai, M., et al. The first archaic *Homo* from Taiwan. Nature Communication 6, 6037 (2015).
3. Zhou, G-X., Yi, G-Y. On the remains from Liuzhou region, Guangxi. Mem. Beijing Nat. His. Mus. 20, 1-21 (1983).
4. Wang, L-H. On the human fossil and stone artefacts found in Baojiyan Cave, Guilin. Acta Anthrpologica Sinica 1, 30-35 (1983).
5. Huang, W-B., Zheng, S-H. An Upper Pleistocene human tooth and mammalian fossils from Zhangwu, Shaanxi. Acta Anthrpologica Sinica 1, 14-17 (1983).
6. Zhao, Z-R., Liu, X-S., Wang, L-H. Human fossils and associated fauna of Jiulengshan Hill, Guangxi. Vertebrata PalAsiatica 19, 45-54 (1981).
7. Yu, J-B. Fossil man and cultural artifacts form Chuandong, Puding County, Guizhou Province. Bulletin of Nanjing University (1), 39-50 (1984).
8. Zheng, L. A fossil human tooth from Zhaotong, Yunnan. Acta Anthrpologica Sinica 4, 105-108 (1985).
9. Sladek, V., Trinkaus, E., Hillson, S. W., et al. The People of the Pavlovian: Skeletal Catalogue and Osteometrics of the Gravettian Fossil Hominids from Dolnı′ Vestonice and Pavlov (Dolnı´ Veˇstonice Studies, 2000).
10. White, T., D., Asfaw, B., DeGusta, D., et al. Pleistocene *Homo sapiens* from Middle Awash, Ethiopia. Nature 423, 742-747 (2003).
11. Rightmire, G. , P., Deacon, H., J. New human teeth from Middle Stone Age deposits at Klasies River, South Africa. J. Hum. Evol. 41, 535-544 (2001).
12. Brauer, G., Mehlman, M., J. Hominid molars from a Middle Stone Age level at the Mumba Rock Shelter, Tanzania. American Journal of Physical Anthropology 75, 69-76 (1988).
